# Applications of 3D models in cholangiocarcinoma

**DOI:** 10.3389/fonc.2025.1598552

**Published:** 2025-07-31

**Authors:** Agata Montagner, Laura Lemberger-Viehmann, Nadine Reitberger, Milena Schmidt, Julia Scheruebl, Eric Pion, Benedikt J. Wagner, Christian Pilarsky, Robert Grützmann, Thiha Aung, Christina Hackl, Silke Haerteis

**Affiliations:** ^1^ Institute for Molecular and Cellular Anatomy, University of Regensburg, Regensburg, Germany; ^2^ Faculty of Applied Healthcare Science, Deggendorf Institute of Technology, Deggendorf, Germany; ^3^ Department of Surgery, University Hospital Regensburg, Regensburg, Germany; ^4^ Division of Surgical Research, University Hospital Erlangen, Erlangen, Germany; ^5^ Department of Surgery, University Hospital Erlangen, Erlangen, Germany

**Keywords:** cancer, cholangiocarcinoma, *in vitro* cancer models, 3D (three-dimensional) models, organoids, tumor spheres, CAM model, personalized medicine

## Abstract

The prognosis for patients diagnosed with cholangiocarcinoma (CCA) is dismal, with an overall 5-year-mortality rate of 80%. Therapeutic approaches for this cancer are very limited and the only curative treatment is total surgical resection despite recent advancements in CCA research. However, only a minority of patients are eligible for surgery due to late-stage diagnosis. Therefore, there is an urgent need to gain a deeper understanding of CCA and to discover new treatments, which can be achieved by utilization and optimization of 3D tumor models. Traditional 2D cell culture is still undeniably important in cancer research, especially for the discovery of biomarkers and drug screening. However, classical 2D tumor models do not represent the tumor biology in its full complexity as they lack the vital interactions between cancer cells, angiogenesis, and tumor microenvironment. In recent years, 3D models, including spheroids, 3D co-culture systems, organoids, tumors-on-a-chip, and the *in vivo* chorioallantoic membrane (CAM) model, have been used for CCA research. These models enable the study of the tumor microenvironment, investigation of metastases, drug development and testing, cholangiocarcinogenesis and personalized therapy. This review summarizes the applications of the different 3D tumor models that have been used for the investigation of CCA. Moreover, the advantages and disadvantages of the different 3D tumor models are discussed, and suggestions for future research possibilities are described. By optimizing 3D models, the gap between basic research findings and clinical applications can be bridged, enabling the discovery of more effective therapies for CCA and other cancers.

## 1 Introduction

**Figure 1:**
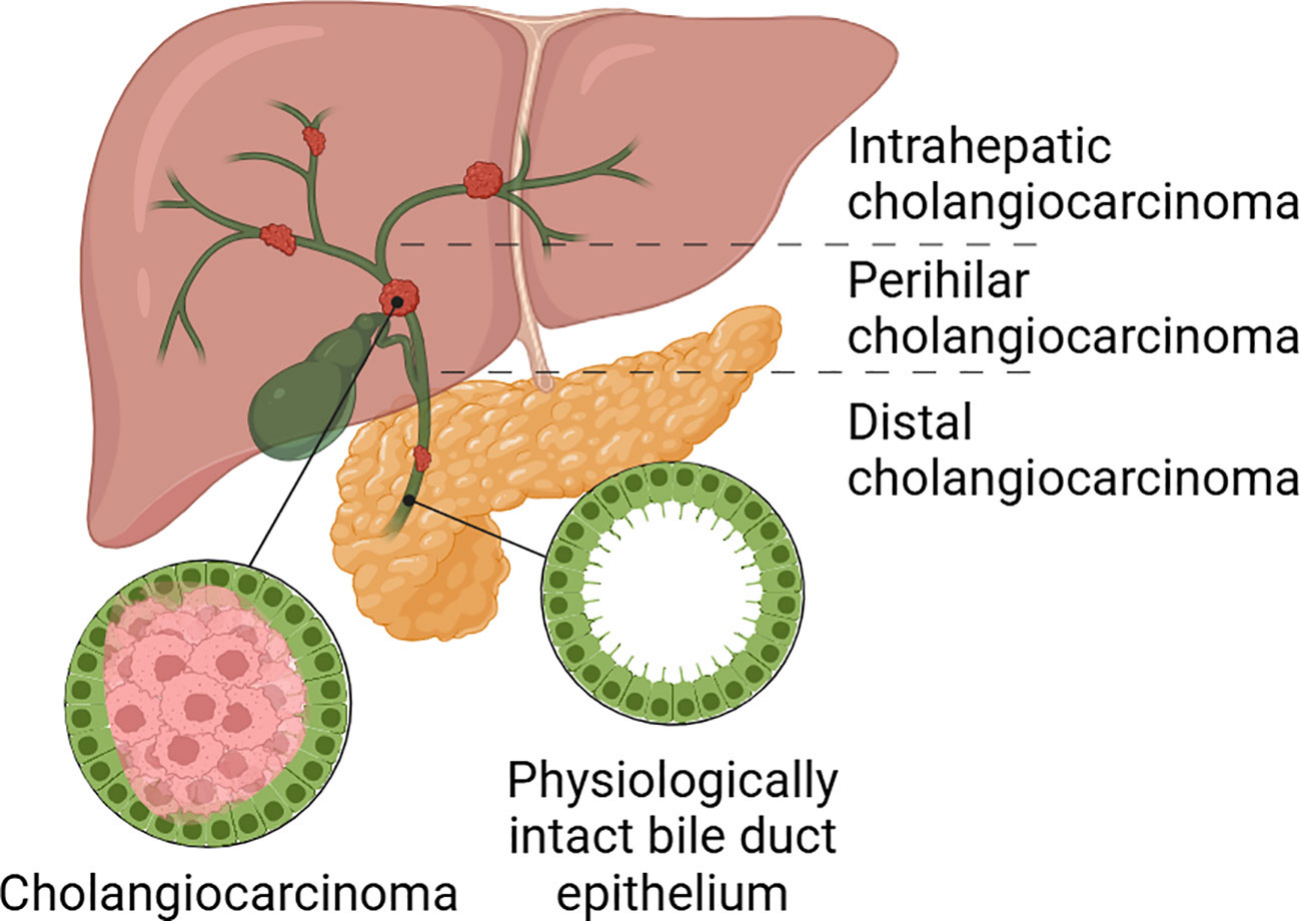
Classification of CCAs according to anatomical location. Cholangiocarcinoma can be divided in intrahepatic, perihilar and distal cholangiocarcinoma. Created with Biorender.com.

Liver cancer is the third leading cause of cancer-related death globally (1). Cholangiocarcinoma (CCA) is the second most common type of primary liver cancer after hepatocellular carcinoma and accounts for 10-15% of primary liver malignancies (1, 2). CCA arises in the biliary tract and is categorized into intrahepatic CCA (iCCA), perihilar CCA, and distal CCA, depending on the affected anatomical location of the bile ducts (**Figure 1**) (3). Often detected at a very late stage of the disease, this malignancy is characterized by a poor outcome (2).

A total surgical resection of the tumor is the only potentially curative treatment for CCA, but many patients are not eligible for surgery (4, 5). Therefore, systemic chemotherapy with gemcitabine, cisplatin and durvalumab is the first treatment option for patients with inoperable or metastasized CCA (2, 6). As an adjuvant therapy, capecitabine has been the only first-line standard therapy since the publication of the BILCAP study (7). Another combination that was studied was gemcitabine and cisplatin, which were investigated for the use as an adjuvant therapy in the ACTICCA study (2). However, most CCAs develop resistance to chemotherapy over time (8).

With these limited therapeutic options, the impending need to understand this malignancy more thoroughly and identify new, effective treatments is evident. For studying carcinogenesis and researching novel therapeutic options, it is fundamental to develop and optimize “modern” models such as three-dimensional (3D) tumor models.

Two-dimensional (2D) cell culture, has been the gold standard for *in vitro* cancer research. However, as 3D models resemble the pathological tumor microenvironment (TME) in a more realistic way (9, 10), they have increasingly gained attention and are now integrated into cancer research across various tumor entities (11–15). 3D tumor models for studying CCA range from simple spheroids and 3D co-culture systems to more advanced approaches like organoids and tumors-on-a-chip and even include the *in vivo* chorioallantoic membrane (CAM) model. This vast array of different 3D tumor models can be utilized for various applications, such as cholangiocarcinogenesis research, drug development, investigating personalized therapies, studying metastasis and TME. This review aims at summarizing the different types and various applications of 3D models that are used to study CCA.

### 1.1 3D models vs. traditional 2D models

2D cell culture, in which cells grow as a monolayer on a flat surface, has been used as a method for over 100 years. The different 2D cell culture methods like monolayer monoculture (16) and co-culture (17, 18) are widely accepted, have been optimized and appear to be cheaper than 3D cell cultures, which makes them more accessible. The growth of cells in a monolayer enables all cells to obtain the same amount of nutrients, thus having a simplified system with a homogenous proliferation. Unfortunately, this type of culture has many disadvantages, with the most important one being the lack of resemblance to the physiological conditions of an organism and the absence of a complex biological microenvironment (9, 19).

**Figure 2:**
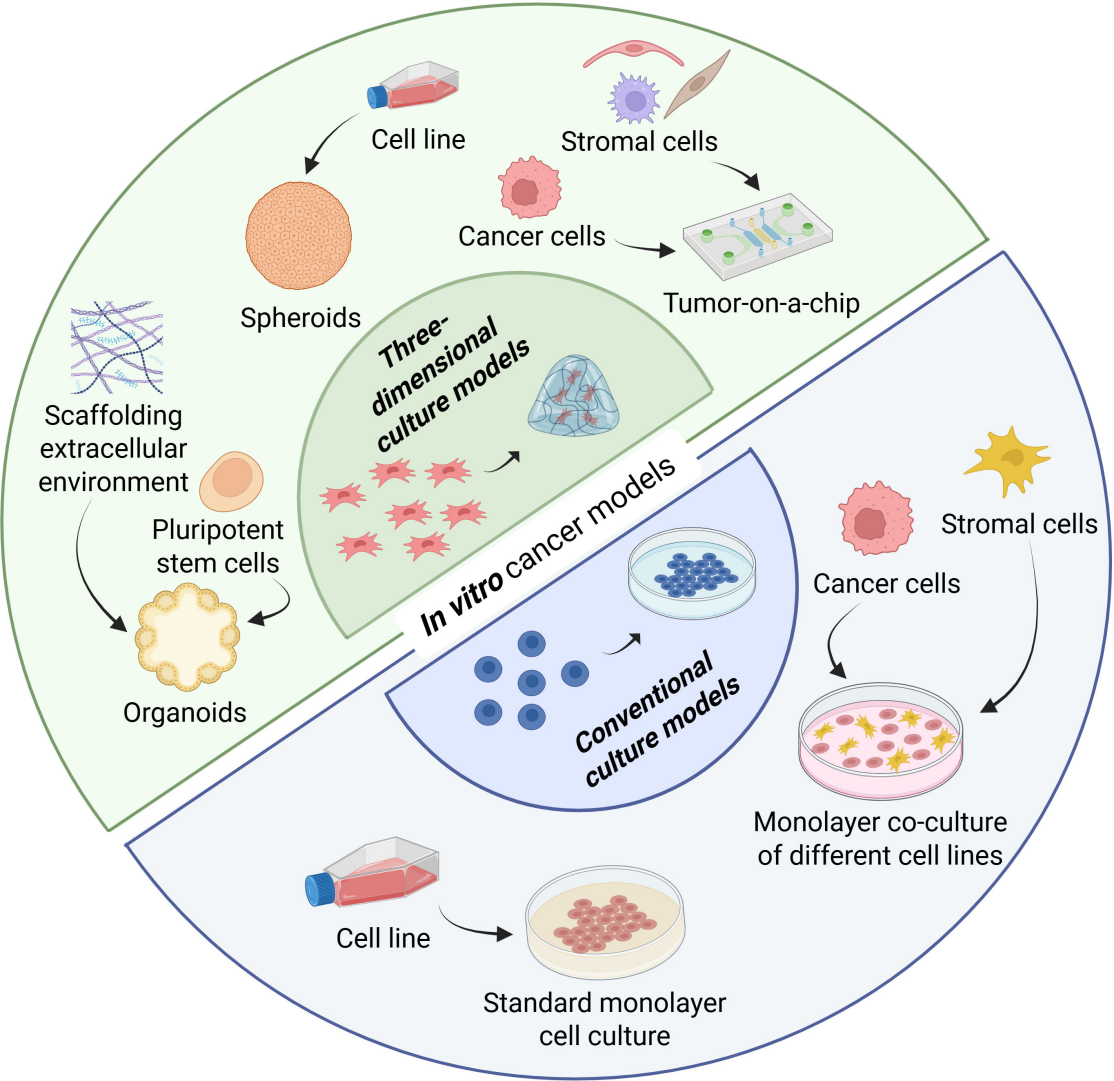
Differences between two-dimensional and three-dimensional *in vitro* tumor models. One of the most diffused *in vitro* tumor models is the standard monolayer cell culture in which only one cell line is cultivated. In the co-culture monolayer method, more than one cell line is cultivated together but still in a two-dimensional system. Spheroids are simple free-floating cellular clusters. Organoids are complex 3D clusters in an arrangement that resembles the function and structure of the organ of origin. Tumors-on-a-chip are advanced models that couple cell culture with microfluidic devices. Created with Biorender.com.

3D cell culture models enable the investigation of cell-cell and cell-matrix interactions, which are of high importance to cancer cells and are connected to physiological and pathological characteristics of the tissue and the affected organs. Signals from the extracellular matrix (ECM) are fundamental for cancer development and progression. Therefore, cancer cells cultivated in 3D showcase a different behavior than cancer cells cultivated in a 2D system (**Figure 2**) (9, 20, 21).

Molecular characterization of CCA models involves various detection methods, each offering distinct advantages depending on whether 2D or 3D culture systems are used. Next generation sequencing is widely applied in 3D CCA models, especially in patient-derived organoids (PDOs) (22–25). Next generation sequencing is used in patient tumor tissue for identifying gene variants in CCA, with the aim of finding possible targeted treatments (26). RNA sequencing is a popular detection method for researching transcriptomics in 3D models (27–29). However, despite its increasing relevance, sequencing remains a relatively new and underrepresented method for the molecular characterization of CCA cell lines (30). To analyze protein expression in 3D models of CCA, immunohistochemistry or immunofluorescence are commonly used, with the added advantage of spatial distribution information (23, 27, 31). In the clinical setting, immunohistochemistry provides important information, aiding diagnosis and prognosis of the patients (32). Immunofluorescence was successfully used to visualize even whole-mount organoids (33). To quantify protein expression levels in the 3D models, without gaining spatial distribution information, western blotting is also possible, even though it requires a slightly more complex dissociation process compared to 2D cell cultures (34–41).

## 2 Different types of 3D tumor models

### 2.1 Spheroids

The generation of multicellular spheroids represents a cornerstone in 3D cell culture technology, offering physiologically relevant *in vitro* models. Spheroids are small dense spherical aggregates of cells that grow in a 3D structure. They can be generated directly from primary cells isolated from tissue from mesenchymal stromal cells with multipotent properties, immortalized or cancer cell lines or from pluripotent stem cells able to differentiate in almost every cell type (42–46). A further classification divides spheroids in homotypic and heterotypic spheroids. Both types usually have distinct regions (a central core with necrotic cells, surrounded by dormant and senescent cells and an outer proliferative zone). For homotypic spheroids, these different regions merely represent the same cancer cell type showing different features, since these spheroids are composed of only one cell line or only one type of primary cells (47). In contrast, heterotypic spheroids include also stromal cells (e.g. fibroblasts, endothelial cells or immune cells), as is more accurately represents the intratumoral heterogeneity (48, 49).

The main characteristics of tumor spheroids are organization of cells in different layers, the presence of a physical barrier created by the deposition of ECM, impaired drug penetration, and gene expression resembling that of *in vivo* tumors, which makes them an ideal method for drug testing (48). Overall, the main advantage of the spheroid culture is the maintenance of phenotypic properties and physical interactions that reflect the tumor biology (20). However, the main disadvantage is the lack of reproducibility caused by extensive variability in spheroid formation due to different factors such as cell type, culture method, medium and cell density (49).

**Figure 3:**
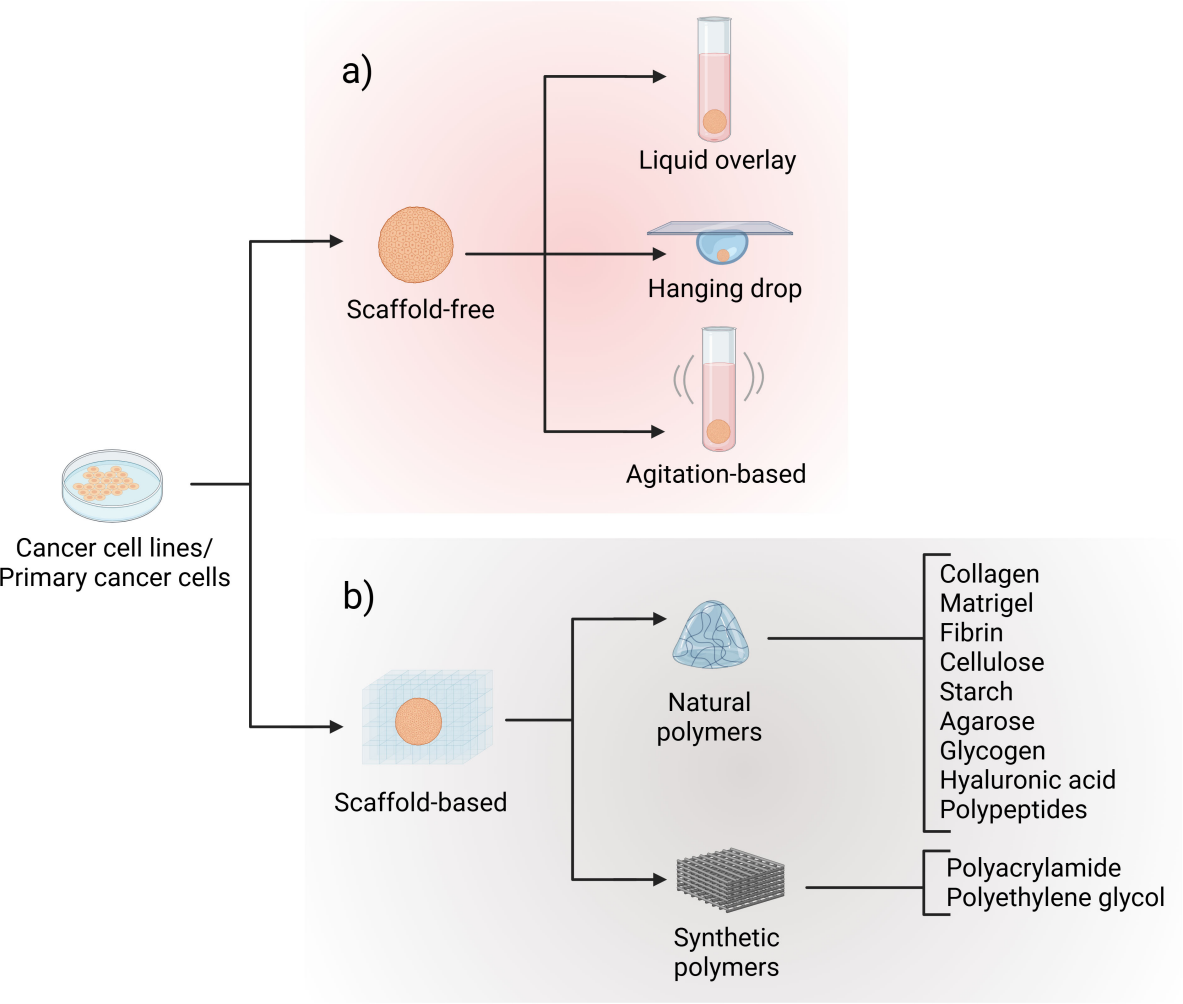
Spheroid formation methodologies. Spheroids can be generated using **(a)** scaffold-free approaches, including liquid overlay, hanging drop, and agitation-based techniques, or **(b)** scaffold-based approaches employing either synthetic or natural polymers. Created with Biorender.com.

Spheroids can be created using commercial cell lines (43), embryonic bodies, as well as different types of tissue such as tumor tissue, neural tissues and mammary glands (42). They can utilize either scaffold-based or scaffold-free methods for their formation (43). Scaffold-free techniques, in which the spheres are not attached to any surface, remain among the most commonly employed approaches, particularly the hanging drop and cell culture plates method. The latter works with an ultralow attachment surface, which inhibits cells to adhere as a monolayer on the surface, forcing them to swim in the culture medium (43). The hanging-drop method uses surface tension and gravity to help single cells agglomerate and form round spheroids in small droplets. By changing the drop size or the number of cells, the size of the spheroids can be controlled easily and at low cost (20). However, this method is technically challenging and has a low-throughput (**Figure 3a**). Scaffold-based approaches incorporate biomaterials like hydrogels that are casted and stabilized in polydimethylsiloxane molds to create cylindrical microwells (44). Hydrogels, used to embed spheroids, mimic the natural environment around cells. Both natural materials (like collagen or Matrigel) and synthetic hydrogels (like GelMA or alginate) affect how spheroids behave - such as how they grow, survive, and release signals (**Figure 3b**). In addition, important hydrogel properties like stiffness, viscoelasticity, and cell-adhesive molecules (e.g. RGD peptides) can influence how cells inside the spheroid respond, especially those near the edge that have actual contact to the material (44). Overall, there is a variety of different techniques for the generation of spheroids (**Supplementary Table S1**). These methodologies enable tailored spheroid generation for diverse applications, from cancer biology to tissue engineering, by integrating the right combination of medium formulation, cell type, and structural support.

### 2.2 Organoids

**Figure 4:**
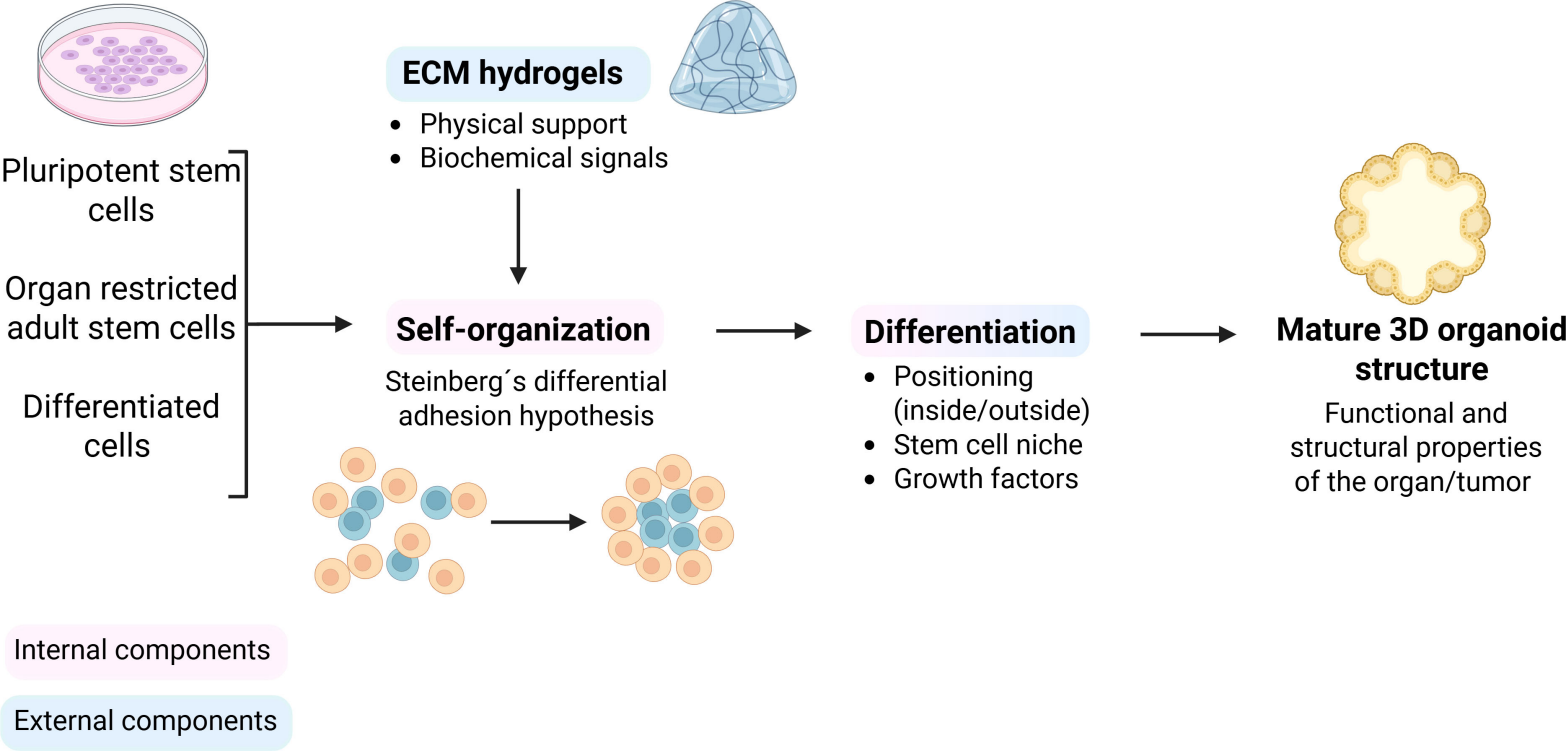
Organoids methodologies. 3D organoids can be generated from various cell sources, including pluripotent stem cells, organ restricted adult stem cells and differentiated cells. With the support of ECM-hydrogels providing physical structure and biochemical cues, cells undergo self-organization based on Steinberg’s differential adhesion hypothesis. This is followed by differentiation, influenced by positioning, stem cell niche and growth factors, ultimately leading to a mature 3D organoid structure that mimics the structural and functional features of the original organ or tumor. Created with Biorender.com.

In contrast to spheroids, which are simple clusters of cells that cannot self-assemble or regenerate (42), organoids represent a self-organizing 3D culture system that bridge the gap between 2D cultures and *in vivo* animal models. These complex 3D structures mimic the functions and architectures of *in vivo* organs (50, 51). The generation of organoids typically begins with adult or pluripotent stem cells - including both embryonic stem cells and induced pluripotent stem cells - which are cultured in a dome or flat gel of a 3D scaffolding matrix (such as Matrigel or Basement Membrane Extract Type 2), beneath a cell culture medium (52). The culture medium must be tissue-specific and is typically supplemented with essential growth factors and signaling proteins, including epidermal growth factor EGF, R-spondin 1 RSPO1, Noggin, and Wnt-3a, which support the maintenance of stemness, proliferation, and lineage specification (53) (**Figure 4**). The efficiency of organoid formation is influenced by the mechanical properties of the matrix, that can be modified to mimic the stiffness of physiological organs (50). In summary, there is an abundant variety of different methodologies for organoid generation (**Supplementary Table S1**). The most used method for cultivating organoids entails a submerged culture in which cells are embedded in ECM gel and submerged by liquid medium. The air-liquid interface culture method uses tissue fragments that are embedded in collagen gel and placed in an inner chamber with the top directly exposed to the air, while the nutrients of a medium placed in an outer chamber diffuse to the inner dish that contains the organoid (54). The most complex culture is the microfluidic device technique, which consists of channels that hold organoids embedded in gel, while fluid (i.e. medium) is pumped through the system (55).

When successfully cultured, organoids typically contain both proliferative and differentiated cells and recapitulate functional and histological characteristics of the donor tissue. Using these protocols, PDOs have been successfully established from a wide range of tumors, including gastrointestinal, pancreatic, colorectal, and hepatic malignancies. Establishment rates vary widely, from less than 20% to over 90%, depending on tumor type, tissue quality, and prior treatments (52). A notable example of organoid generation from adult tissue includes the development of intrahepatic cholangiocyte organoids, first described by Huch et al. (56), where Lgr5^+^ liver stem cells were cultured under conditions enriched with growth factors to yield biliary epithelial-like structures. These organoids expressed specific cholangiocyte markers such as Epithelial Cell Adhesion Molecule, SRY-Box Transcription Factor 9 and Keratin7/19, and demonstrated biliary function, including Multidrug Resistance Protein 1-mediated Rhodamine123 transport into the organoid lumen (56). Parallel approaches enabled the long-term culture of hepatocyte organoids using hepatocyte-specific medium, which resulted in the expression of hepatic markers such as albumin and HNF4α (57). Collectively, these methodological advances have enabled the establishment of diverse organoid systems - from brain and pancreas to colon and liver - that serve as robust platforms for disease modeling, regenerative medicine, and precision oncology.

### 2.3 Tumors-on-a-chip

Tumors-on-a-chip are one of the most advanced and complicated *in vitro* models. These devices mimic the organization of the cells inside a particular tissue and thereby aim to replicate the functionality of an organ. This model combines 3D cell culture with microfluidic devices (58). 3D channels and chambers are 3D-printed by using flexible polymers. These spaces are then filled with cells inside hydrogel or perfused with different types of fluids. Not only tumor cells can be seeded inside this model but also cells of the stroma such as monocytes, endothelial cells, fibroblasts, hepatocytes and hepatic stellate (HS) cells. All in all, this model allows to mimic the primary tumor and the metastatic microenvironment due to its modular design and highly controlled physiochemical properties (59).

### 2.4 Chorioallantoic membrane model

In contrast to the previously mentioned models which are all *in vitro* tumor models, the CAM model is an *in vivo* 3D tumor model. It is based on the CAM, an extraembryonic, highly vascularized membrane that develops within fertilized chicken eggs and is responsible for gas exchange of the chick embryo (60, 61). Both commercial cell lines and tumor tissue can be grafted onto the CAM for subsequent cultivation *in vivo* (*in ovo*). During the cultivation period on the CAM, many different aspects of the tumor biology can be monitored, such as tumor growth, angiogenesis and metastasis (62). The model is also an ideal platform for the testing of therapeutics (63), with the possibility to inject drugs intravenously due to the high vascularization of the CAM (64). 3D models can also be combined, e.g. the CAM model and tumor spheres. In a recently published study, Wagner et al. validated the successful cultivation of tumor spheres on the CAM, demonstrating their potential to monitor therapeutic response and disease progression (65). These studies highlight the crucial role of the CAM model in developing and testing novel therapeutic approaches. Moreover, this model contributes greatly to reducing animal testing in accordance with the 3R principle (“Reduce, Replace, Refine”) (66). In cancer research, the CAM model is regarded as an intermediate step for the translation of preclinical research results into clinical applications (67).

## 3 Use of 3D tumor models for research of cholangiocarcinoma

**Figure 5:**
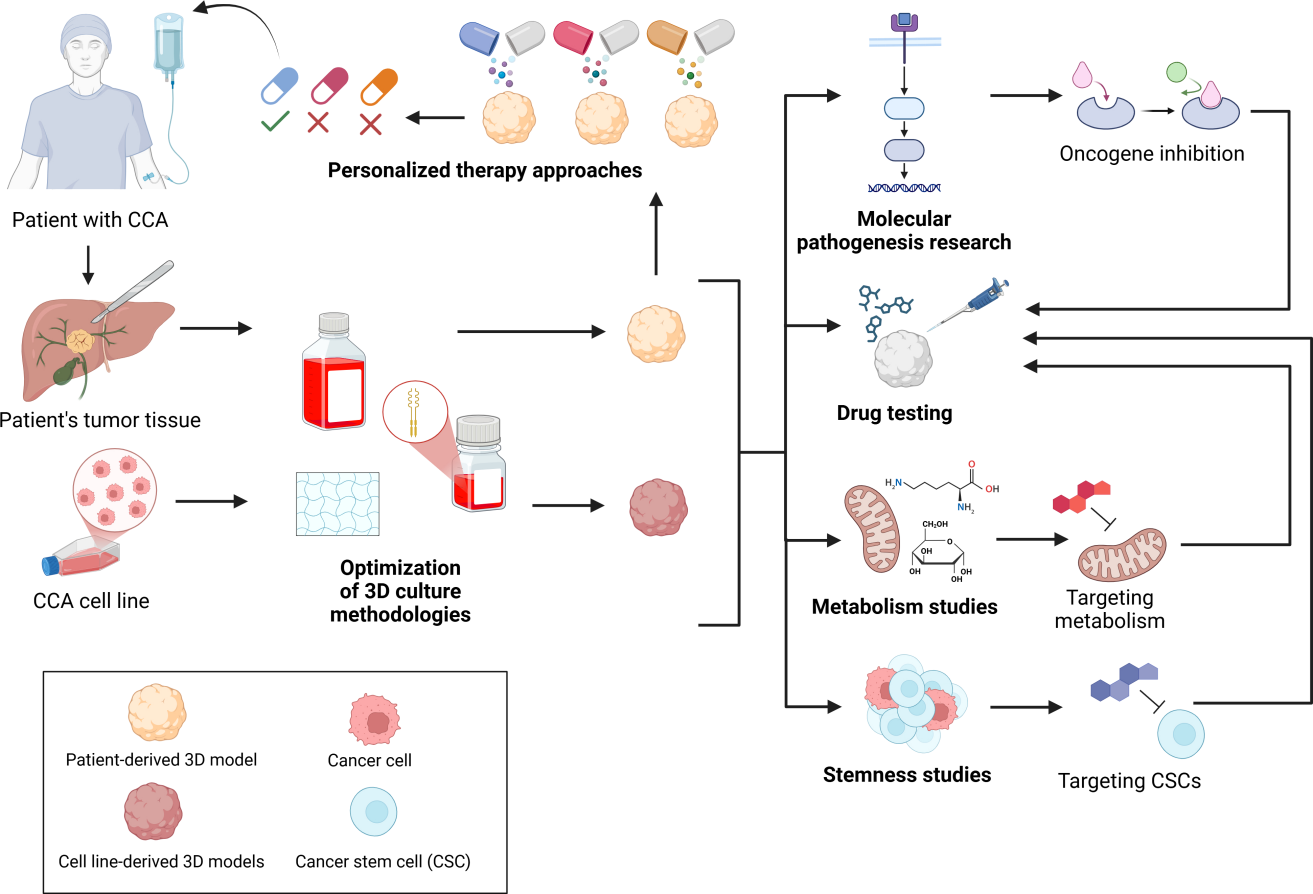
Applications of spheroids and organoids in cholangiocarcinoma research. Tumor tissue from CCA patients or established CCA cell lines is used to generate 3D models through Optimized culture methodologies. These 3D models support studies regarding Molecular pathogenesis, Metabolism, Stemness, Drug Testing and Personalized therapy approaches. Created with Biorender.com.

Preclinical research is crucial for gaining a deeper understanding of CCA and enhancing therapeutic outcome. This involves the development and management of various experimental models, including traditional *in vitro* assays with primary or commercial cell lines cultured in 2D, 3D spheroids and organoids, 3D co-culture of cancer cells with microenvironmental cells, advanced tumor-on-a-chip systems, and 3D *in vivo* models with engrafted tumor tissue. Each model offers distinct advantages and limitations (see **Table 1**) (10). Since traditional 2D culture presents altered signaling and migration (68), increased proliferation and different chemosensitivity compared to testing in patients (9, 69), there is an urgent need to implement more 3D tumor models in cancer research. 3D models can be utilized for various applications in CCA research, since they offer the possibility to accurately reproduce tumor biology, investigate cell interactions and to develop personalized therapies (**Figure 5**).

**Table 1:** Summary of the most important advantages and disadvantages of the various culture models.

Model type	Model subtype	Advantages	Disadvantages	References
2D models	Classical	Good standardization and reproducibility Simple handling Controllable environment Large scale testing Cost-effective	No ECM Unrealistic cell-cell interactions	(9, 10, 19, 269)
	2D co-culture	Intercellular interactions Flexible cell composition	Complexity of analysis Varying growth requirements	(17, 18, 270)
3D *in vitro* models	Spheroids	Mimic cell-cell signaling of *in vivo* tissue Heterotypic spheroids represent intratumoral heterogeneity More affordable than organoids	Lack of standardized techniques and assays Lack of reproducibility	(48, 49)
	Organoids	Mimic physiology of *in vivo* organs Possibility of personalized medicine applications Genetic stability maintained from parental cells	High cost of growth factors and nutrients Lack of reproducibility	(51, 54, 266)
	3D co-culture	Intercellular interactions in the presence of ECM	Lack of standardization	(17, 18)
	Tumors-on-a-chip	Mimic TME Highly controlled physiochemical properties Real-time monitoring	Lack of user-friendly methods High cost of bioprinting	(58, 59, 245, 248)
3D *in vivo* models	CAM model	Potential for personalized therapy Conforms to the 3R principle Analysis of angiogenesis in real time Cost-effective	Sensitive to environmental influences Limited observation time due to fast embryo development	(62, 263)

2D cell culture is a method that has been used for more than a century. Therefore, it has been highly optimized and its cost are relatively low. A major advantage of 3D *in vitro* models is the ability to mimic the important TME, thus they represent a good option especially for drug testing and (personalized) therapy. Unfortunately, such 3D models lack standardization and reproducibility and are usually expensive.

### 3.1 Spheroids/tumor spheres in cholangiocarcinoma

Recently, there has been an increased inclusion of these 3D tumor models for the study of CCA with a focus on molecular mechanisms, biomarkers, cancer stem cells (CSCs), tumor growth and TME. Spheroids are three-dimensional aggregates of cancer cells cultured under non-adherent conditions, serving as models to study tumor behavior and drug responses. Tumor spheres, on the other hand, are derived from single tumor cells cultured in serum-free media supplemented with growth factors, primarily used to enrich and study cancer stem-like cells (70). Therefore, CCA-derived spheroids and tumor spheres may provide a promising experimental approach for future CCA research.

#### 3.1.1 Spheroids as a model for cancer stemness studies

One of the most widespread applications of the spheroid model is the study of stemness, the ability of CSCs for self-renewal and differentiation (71), through the spheroid/sphere formation assay.

The role of CSCs in cholangiocarcinoma progression has been extensively studied, with numerous reports highlighting key molecular regulators that drive stemness, metastasis, and therapy resistance. Cardinale et al. investigated the tumorigenic potential of different CSC populations and they generated stromal tumors or epithelial tumors depending on the microenvironment (72). Arnoletti et al. demonstrated that CSCs form immune cell clusters that enhance metastatic potential, emphasizing their crucial role in CCA progression (73). Panawan et al. established a CCA-like cancer stem cell line that is able to form tumor spheres, and thereby serves as a representative model for CSCs (74). Wang et al. used a fluorescent reporter system to track and isolate CSCs in CCA, revealing a bidirectional crosstalk between CSCs and macrophages that facilitates CSC renewal and macrophage polarization in 3D cultures (75). Additionally, Yogo et al. found that inhibiting dopamine receptor D1 signaling promotes CSC-like growth in bile duct cancer cells (76). This further underscores the intricate regulatory networks sustaining CSC populations.

A number of studies focused on investigating the role of various molecules within the CSCs in CCA, especially regarding their role in tumor sphere formation. This included four newly identified stemness associated genes (SDHAF2, MRPS34, MRPL11, COX8A) (77), the immune regulator Tripartite motif-containing 29 (78), the GTPase RAB6A (79), the deubiquitinating enzyme Ubiquitin Specific Peptidase 10 (80), Keratin18 (81) and the ganglioside GD2 (82). In addition, non-coding RNAs have been studied as they are implicated in maintaining stemness and oncogenic properties in CCA. It was shown that miR-200b/c regulates tumorigenic and metastatic properties by targeting Rho-kinase 2 and SUZ12 (83), while the long non-coding RNA PKD2-2–3 promotes drug resistance and CSC marker expression (84). In line with findings regarding the molecular pathology of cancer stem cells and resistance, Shi et al. demonstrated that Y-box binding protein 1 enhances stemness and cisplatin resistance in iCCA by activating the AKT/β-catenin pathway and promoting spheroid formation (85). Similarly, Sugiura et al. reported that inhibiting the Yes-Associated Protein, through siRNAs or verteporfin, reduces spheroid formation and anoikis (a form of programmed cell death) resistance in iCCA, further supporting the role of molecular pathways in CSC maintenance (86).

Together, these studies illustrate the complexity of CSC regulation in CCA, highlighting critical molecular pathways and potential therapeutic interventions aimed at disrupting CSC-driven tumorigenesis.

#### 3.1.2 Drug testing in spheroids

3D spheroid models have become an essential tool in drug discovery. By resembling the TME, spheroids allow for a more accurate assessment of drug penetration, resistance mechanisms, and therapeutic efficacy. The search for novel therapeutic compounds for CCA using spheroids has led to promising discoveries, including also natural compounds. The effects of Resveratrol (polyphenolic compound produced by plants) were investigated in 3D CCA spheroid models, demonstrating significant inhibition of cell growth, reinforcing its potential as a therapeutic agent (87, 88). Similarly, Pant et al. explored an innovative treatment strategy using butyrate, a fatty acid produced through fermentation of fibers by gut bacteria (89). Pang et al. isolated new steroidal glycosides from the Trillium tschonoskii rhizome that were able to suppress sphere formation (90). In these studies, 3D CCA spheroids were created using CCA cell lines, and various concentrations of therapeutic compounds were tested to assess their effects on spheroid growth, size, proliferation, and viability.

Targeted therapies have emerged as promising approaches for CCA focusing on key signaling pathways, metabolic regulators, and immune-based strategies. Several studies have identified critical molecular targets that regulate tumor growth and therapeutic resistance. Possible targeted therapy approaches for CCA, like inhibiting the tyrosine kinase with Ceritinib (91), or the inhibition of the cyclin-dependent kinases 4 and 6 (92), of cell division cycle protein 20 (93), of long non-coding RNA LINC00665 (94), of mTORC1 (95), of CCR5 (96), and of protein SUMOylation (97), were studied using spheroids. Targeting CSC-associated pathways has also emerged as a promising therapeutic strategy. It was demonstrated that targeting the oncogene CXCR7 and the tumor suppressor FBXO31 could be effective approaches (98, 99). In these studies, the inhibition of these molecules was able to suppress spheroid growth.

Inhibiting drug resistance mechanisms has also been a focus of recent studies, since chemoresistance is still a major problem in CCA (8). Some candidate drugs possibly able to target chemoresistance in CCA were discovered using the spheroid model like insulin and insulin-like growth factor 1 receptor inhibitors (100), the cardiac glycoside Ouabain (101), the natural compound Saikosaponin-a (102) and the antibiotic Metronidazole (103). Sphere formation assay was also utilized in the realm of chemoresistance studies for the development of new cell lines that present a similar sensitivity as the *in vivo* tumor. Two new iCCA cells lines were created, one highlighting chemotherapy resistance (104) and the other one that could serve in the future as tool for drug testing in preclinical research (105).

Combination therapies have also shown efficacy in preclinical models. Fu et al. demonstrated that verteporfin, a photodynamic drug (is activated by light), reduced tumor growth, apoptosis, and stemness; and when combined with anti- programmed cell death 1, significantly lowered tumor burden in CCA mouse models (106). While Bai et al. showed that the combination of Hinokitiol and palbociclib had a strong inhibition in tumor sphere formation (107). Another drug combination that effectively induced cell death and decreased proliferation in CCA spheroids was the combined treatment of CDK4/6 (key regulators of cell cycle progression) inhibitor palbociclib with the Smac mimetic LCL161(a Cellular Inhibitor of Apoptosis Proteins 1 and 2 antagonist) (108).

Immune-based therapies have also proven effective in disrupting CCA spheroids and boosting immune responses. Phanthaphol et al. developed CAR-T cells targeting integrin αvβ6, demonstrating high cytotoxicity against CCA spheroids (109). Suwanchiwasiri et al. further expanded on this strategy by designing a bispecific T cell engager targeting integrin αvβ6 and CD3, demonstrating enhanced T cell activation and cytotoxicity (110). Recently, Phanthaphol et al. tested fifth-generation CAR-T cells that target the PD-1/PD-L1 pathway, exerting a high cytotoxicity and penetrating deep into spheroids (111). Additionally, CAR-T cells against CD133 were created and they lysed CCA spheroids almost completely (112). Afterwards, T cells were engineered to secrete a bispecific T-cell engager targeting CD133, that was effective in disrupting spheroids (113). Supimon et al. created anti-MUC1 CAR-T cells, which significantly enhanced cytotoxicity against Mucin1-expressing CCA cells (114). Lastly, a novel treatment strategy involving induced pluripotent stem cell-derived natural killer cells was explored for cholangiocarcinoma (115). These studies underline the importance of therapeutic strategies that target the immune microenvironment in CCA.

Collectively, these findings highlight the diverse and evolving treatment options in CCA, emphasizing the potential of molecular inhibitors, metabolic regulators, and immune-based strategies to improve treatment outcomes. By providing a more physiologically relevant platform, spheroids facilitate the identification of novel therapeutics and combination treatments, ultimately advancing the development of more effective treatment strategies for CCA.

#### 3.1.3 Study of molecular pathogenesis in spheroids

Molecular pathology studies have identified key oncogenes and tumor suppressors that regulate CCA progression, stemness, and therapeutic resistance using spheroids.

Oncogenic pathways driving cholangiocarcinogenesis have been extensively studied. Hasegawa et al. demonstrated that inhibiting fatty acid desaturase 2 significantly reduced tumorigenicity, cell proliferation, migration, and sphere formation in CCA cells (116). Likewise, Correnti et al. showed that SerpinB3 overexpression enhances invasion and sphere formation in CCA stem-like cells, correlating with poor prognosis (117). Other studies focused on tumor-promoting genes, with Singsuksawat (118), Carotenuto et al. (119) and Lobe (120) identifying the hormonal regulator E26 transformation-specific variant 4 (118), the transcribed-ultraconserved region uc.158- (119) and the transcription factor zinc finger E-box-binding homeobox 1 as potential therapeutic targets (120). Also, the thyroid hormone T3 was shown to possibly have a pro-tumorigenic effect as CCA cells treated long term with the hormone formed spheroids in bigger size and quantity, in the presence of gemcitabine (121).

Conversely, tumor suppressors have also been identified as key regulators of CCA. Yoshino et al. found that AT-rich interactive domain-containing protein 1A deficiency promotes tumor growth and stemness, correlating with poor prognosis (122). Additionally, Shu et al. identified Numb as potential therapeutic target (123). Tumor suppressing properties were also investigated in non-coding RNAs. A novel tumor suppressor role was discovered for microRNA-876 (124), while microRNA let-7c presented a dual function in CCA, inhibiting tumor growth but promoting distant metastasis (125).

Several studies have explored molecules in oncogenic pathways that could be targeted in future treatment approaches. Puthdee et al. demonstrated that blocking TGF-β signaling effectively prevents LIN28B-induced metastasis in CCA (126). Similarly, Romanzi et al. showed that angiopoietin-2 and vascular endothelial growth factor stimulate tumor invasion, while their inhibitors suppress this effect (127).

Together, these findings emphasize the complex interplay between oncogenes and tumor suppressors in CCA, highlighting key molecular targets for potential therapeutic intervention.

#### 3.1.4 Metabolism studies in spheroids

Research on the metabolic regulation and tumor biology of CCA has demonstrated that 3D spheroid models provide crucial insights into underlying mechanisms.

Phukhum et al. created spheroids from well differentiated CCA cell lines resulting in a hypoxic and oxidative microenvironment (129). Similarly, Mischiati et al., showed that spheroids presented an altered protein expression, a metabolic rewiring, and an enhanced mitochondrial respiration (130). Specifically, CCA spheroids exhibited increased iron content, heightened oxidative stress, and elevated CSC markers (131). Moreover, CCA cells were grown as spheres, they showcased an enhanced mitochondrial respiration (132). Moreover, altered fatty acid metabolism in iCCA promoted a stem-like phenotype, and inhibiting fatty acid synthase significantly reduced sphere formation and tumor growth (133).

Another therapeutic approach targeting CCA metabolism was explored by Di Matteo et al. (134). Their research based on the fact that the farnesoid X receptor, a nuclear metabolic receptor, is strongly downregulated in iCCA. Thus, they tested an farnesoid X receptor agonist obeticholic acid in spheroid models and found a significant inhibition of cell proliferation, migration, and spheroid formation, with higher concentrations (up to 2 µM) completely prevented spheroid formation. These findings highlight obeticholic acid as a potential therapeutic compound for CCA (134). Lastly, Ciufolini et al. examined metabolic differences between 2D and 3D cultures of iCCA cell lines, revealing significant metabolic shifts in 3D models regarding central carbon and glutathione metabolism (135). In conclusion, this emphasizes the relevance of 3D spheroid models for accurately assessing metabolic processes and developing targeted therapies.

#### 3.1.5 Spheroids for investigation of CCA heterogeneity and developmental mechanisms

Yang et al. (136) used the spheroid model to compare the growth between iCCA and extrahepatic (eCCA). Even though the size of the two types of spheroids was similar, the mechanism of spheroid formation differed. iCCA developed spheroids from single cell proliferation, while eCCA developed spheroids through the aggregation of cells (136). Recently, it has been inferred that bipotent hepatic progenitor cells (cells that can differentiate into either hepatocytes or cholangiocytes) could be a cell lineage with the ability to potentially generate cholangiocarcinoma. Xu et al. (137) selected a population of CCA cells through fluorescence-activated cell sorting. Through colony formation, cell proliferation, and spheroid formation assays, it was confirmed that this cell population presented indeed features of hepatic progenitor cells (137). Furthermore, the infection of the biliary duct with the human liver fluke *Opisthorchis viverrini*, has been identified as environmental risk factors for CCA. Cholangiocyte spheroids were treated with excretory-secretory products from flukes and afterwards subjected to transcriptomic profiling for better understanding liver fluke infection as a risk factor for CCA (138).

### 3.2 Organoids of cholangiocarcinoma

Organoids are being increasingly integrated into cancer research for a wide range of tumor entities (139). Technologies regarding bile duct organoids have continuously advanced in the last years (140). Possible applications of CCA organoids include drug screening, molecular pathogenesis, metabolism studies and research on risk factors that influence the development of this malignancy.

#### 3.2.1 Drug screening and testing in organoids

One of the most important applications of organoids is drug testing (141). Fidelity in drug testing means that different generations of organoids have consistent testing results, organoids’ results are similar to patients’ and consistent results are present in technical repeats (142). Moreover, for organoids to be reliable preclinical models, it is essential that they resemble the original tumor not only morphologically, but also on a molecular level. This includes the retention of key driver mutations, copy number alterations, and gene expression patterns found in the patient`s tumor tissue. Organoids usually display high fidelity and their genomes are stable, even after multiple passages (142, 143). An often overlooked factor is intratumoral heterogeneity. While bulk sequencing mainly captures dominant clones, only organoids derived from multiple tumor regions or single cells can reflect full clonal diversity. However, most CCA studies lack such analyses (144). The composition of the culture medium also plays a crucial role in maintaining tumor fidelity. In the context of CCA, the medium may include hepatocyte growth factor to promote hepatobiliary growth (90, 145).

Several studies have successfully applied CCA organoids for drug screening. Koch et al. (27) used iCCA PDOs for testing the multikinase inhibitor sorafenib. A pipeline was created to evaluate the effect of the drug including measurement of organoid size, immunohistochemistry and RNA sequencing as a method for future patient-specific drug screening (27). Recently, Kaldjob-Heinrich et al. tested Namodenoson (an adenosine A3 receptor agonist) on CCA cell lines and on a CCA PDO (128). Not only single drugs but also combination treatment can be tested with the CCA organoid model. The triple therapy consisting of gemcitabine, cisplatin and LCL161 (second mitochondrial-derived activator of caspases mimetic) was tested in CCA PDOs and was seen as an effective synergic combination, preventing multidrug resistance (146). The combination of oxaliplatin and palbociclib caused drug synergism in iCCA PDOs (147). Treatment with gemcitabine and the HER2 inhibitor lapatinib also resulted in a synergistic effect, when tested in CCA PDOs (148). Bai et al. tested the combination of Hinokitiol, a phytochemical from cypress trees, with the CDK-inhibitor palbociclib which significantly inhibited the formation of organoids (107). Other natural compounds that were effective against iCCA organoids were steroidal glycosides extracted from *Trillium tschonoskii* rhizomes (90). Further, Cavalloro et al. identified and isolated a metabolite from a lichen called usnic acid with anti-cancer properties, that when tested in ICCA organoids led to a decreased viability and modified morphology (145).

Another highly relevant therapeutic strategy is immunotherapy. An example of immunotherapy that has especially attracted a lot of attention lately is CAR-T cell therapy (149). Qiao et al. established a co-culture system with digested CCA organoids and autologous CAR-T cells with knockdown of six inhibitory membrane proteins, that resulted in elevated apoptosis (150). In the realm of immunotherapy antibody-conjugates for antibody-targeted delivery of drugs are also included. Hosni et al. tested the conjugate sacituzumab govitecan on iCCA organoids and all PDOs presented growth inhibition and altered organoid morphology (151).

Photodynamic therapy (PDT), a minimally invasive treatment that combines a photosensitizing agent with light to produce reactive oxygen species and induce cancer cell death (152), was also investigated using the CCA organoid model. CCA PDOs were treated with polyhematoporphyrin-mediated PDT and sulfasalazine individually. The combination of both treatments led to a significant increase in apoptosis, suggesting that this approach could be a potential future treatment for CCA (153). Huang et al. (154) tested a different combination treatment of PDT including surufatinib (a new multikinase inhibitor) on CCA organoids. The combination of surafatinib and PDT effectively killed CCA organoids (154).

Organoids can also be used for screening drug panels. Feng et al. performed high-throughput screening on iCCA to find new therapeutic strategies, resulting in different drug sensitivities throughout different tumor mutations organoids (155). Drug screening was also performed in iCCA PDOs with different BRAF variants, to associate BRAF variants with targeted therapy results (156). While Wang et al. focused on establishing an eCCA PDO for drug screening, after confirming the comparability between the primary tumor and the organoid (157). Through drug screening, interesting compounds were discovered having significant suppressive role in CCA organoids such as the antifungal drugs amorolfine and fenticonazole (25). After extensive drug testing on CCA and hepatocellular carcinoma PDOs, Li et al. discovered that some candidates, e.g. histone deacetylases inhibitors, were effective in the majority of organoids, making them possible candidates for pan-effective treatments in the future (158). Screening of various kinase inhibitors revealed many compounds that could also serve as pan-effective inhibitors, based on the finding that CCA organoids presented a similar increase in the activity of multiple kinases (159). The multikinase inhibitor Sorafenib was discovered as being efficient in decreasing the size of organoids that lacked the tumor suppressor Cullin3 (160). Overall, the organoid emerges as a good model to both screen panels of drugs for discovering new effective compounds for cholangiocarcinoma as well as to test drugs already used in the clinic to investigate their molecular mechanism further and find new successful drug combinations.

#### 3.2.2 Personalized therapy approaches using organoids

One of the most advanced applications of the organoid model are personalized therapy approaches, allowing for the direct translation of laboratory results to patient treatment. The first step in this process is the generation of PDOs that derive from primary material. Broutier et al. (24) were successful in establishing organoids from primary CCA cells. These organoids maintained the characteristics of the individual patients (histology and bulk mRNA expression pattern) even after prolonged *in vitro* expansion. Moreover, *in vitro* testing of different compounds was performed on these organoids and ERK inhibition was uncovered as a potential therapeutic strategy for cholangiocarcinoma (24). Maier et al. (31) also established CCA organoids from surgical resection material, with an impressive organoid from a patient with metastatic iCCA that was successfully kept in culture for 103.3 weeks. To evaluate their tumorigenicity, PDO xenografts were created by injecting immunodeficient mice with PDOs. The xenograft tumors presented similar histological features compared to their primary tumors (31). Wang et al. (161) performed personalized drug screening in an iCCA organoid to compare the effect of conversion therapy on organoid to patient testing. The results of the organoid drug testing were comparable with those of treatment of the respective patient (161). Ren et al. (162) screened common chemotherapeutic drugs on CCA PDOs and the testing was repeated on CCA PDO xenografts: the response of treatment in the majority of the patients was comparable to the response of the respective PDOs. The overarching goal would be to predict in advance the response of CCA to chemotherapeutic treatments (162). A recent example for application of personalized treatment using the organoid model involved the establishment of PDOs from a patient with perihilar CCA which was then subjected to drug screening, showing sensitivity to gemcitabine and cisplatin. Therefore, the drug combination was used in the patient together with toripalimab and lenvatinib. The patient experienced no recurrence a year after surgery (163). Personalized therapy is one the most ambitious goals in oncology. Using a 3D patient-derived model, like the PDO, to predict the efficacy of the treatment for a specific patient could greatly assist the tumor board in making informed therapeutic decisions.

#### 3.2.3 Study of molecular pathogenesis in organoids

The organoid model has also been extensively used to investigate CCA key driver mutations as well as for the exploration of the role of less researched molecules in CCA tumorigenesis. The most distinctive key driver alterations of iCCA include Fibroblast Growth Factor Receptor 2 (FGFR2) fusions and isocitrate dehydrogenase (IDH) 1 mutations (164). In contrast, eCCA is typically characterized by KRAS and SMAD4 mutations (165), thus molecularly more similar to pancreatic cancer (166). Despite advances in molecular profiling, there are two main challenges in targeted therapies in CCA patients. Firstly, many patients lack targetable mutations – for example, FGFR2 fusions/rearrangements are present in only 8-14% of CCA patients (167). Secondly, many patients develop resistance even to the targeted therapies (168). Therefore, there are currently very limited targeted therapy approaches in CCA (169). To overcome these challenges, further investigation into the most prevalent and functionally relevant mutations in CCA is crucial and a possible model for this application is the organoid.

Fujiwara et al. (170) used intrahepatic biliary organoids with mutated IDH1 (an enzyme involved in the Krebs cycle), to study the consequence of this mutation. The mutation was able to increase the organoid formation and altered cell metabolism (170). iCCA PDOs with the wild type and the mutated IDH1 were treated with dasatinib in combination with M2698 (inhibitor of p70 S6 kinase and AKT) to block phosphorylated ribosomal protein S6. The levels of phosphorylated ribosomal protein S6 decreased in PDOs with IDH mutation, while organoids with wild type IDH did not respond to the treatment (171). Regarding the fusion of the growth factor FGFR2, liver organoids with the FGFR2 fusion protein were used to discover that Ras-Erk is a necessary pathway downstream to FGFR2 fusion protein (172). Hogenson et al. (173) identified a new FGFR2-KIF5C chromosomal fusion in CCA patient intrahepatic metastasis and the fusion was also preserved in the PDO. The efficacy of the FGFR inhibitor tested in this PDO was higher compared to a general multikinase inhibitor Lenvatinib. Thus, underlining the importance of sequence in directing possible treatments (173).

However, other therapeutic targets for patients who do not present these distinctive CCA mutations have to be explored. Thanks to the organoid model, many oncogenes have been explored regarding their function in cholangiocarcinoma, including Heat Shock Factor 1 (174), the enzyme Protein arginine-methyltransferase 5 (175), the ribosomal protein S6 (176), dopamine receptor D1 (76), the pathway LTβ/NIK/RelB (177), the enzyme Glycogen Phosphorylase Brain Form (178), the enzyme Aspartate Beta-Hydroxylase (179) and the transporter Solute Carrier Family 16 Member 3 (180).

Non-coding RNAs, like microRNA-21 have also an oncogenic function in many different malignancies (181). Using the CCA organoid model, the predictive role of microRNA-21 regarding the effect of chaperone HSP90 inhibition (182) and the involvement of long non-coding RNA titin-antisense RNA1 in CCA regulation was investigated (183). Finally, DNA networks like neutrophil extracellular traps were also researched in connection to CCA (184).

Besides studying oncogenes in the CCA pathogenesis, also tumor suppressors, like the deubiquitinating enzyme BAP1 (185), the CDK4/6 inhibitor *Ink4* (186) and the non-coding circular RNA circPCSK6 were discovered using CCA organoids (187). Moreover, organoids were utilized for target testing approaches, for example with the new tyrosine kinase inhibitor called NTRC 0652–0, showing cytotoxic effects (188). Cholangiocarcinoma organoids were also treated with antagonists against the voltage-dependent anion-selective channel isoform 1, a protein that regulates apoptosis, resulting in decreased cell survival (189). Aided by the iCCA organoid model, it was discovered that the downregulation of the multispan transmembrane protein MAL2 either through knockdown or using the MAL2 inhibitor Sarizotan decreased the resistance to cisplatin (190).

For studies investigating potential therapeutic targets, it is essential that the model maintains the molecular characteristics of the tumor. Therefore, stability and precision of the organoid makes them an excellent model for this application (142, 143).

#### 3.2.4 Metabolism studies in organoids

CCA organoids have previously been used for metabolic studies. Yoshikawa et al. (40) showed that CCA organoids cultured in absence of glucose were smaller and had low proliferative activity, but the expression of different stem cell markers increased compared to organoids cultivated in the presence of glucose. Not only did the organoids grown without glucose have an increased stem cell phenotype, but also became less sensitive to gemcitabine (40). Lysine deprivation or treatment with the Lysyl‐tRNA Synthetase inhibitor cladosporin in CCA PDOs resulted in significant inhibition of organoid growth and organoid initiation from single cells (191). Li et al. (192) focused on the study of mitochondrial fusion, a phenomenon that could lead to a metabolic advantage in CCA. Mitochondrial fusion was discovered to be increased in CCA organoids and promoted the growth of organoids. Knocking down the fusion regulator genes, Optic atrophy 1 or Mitofusin 1, resulted in a change of mitochondrial morphology and growth inhibition (192). Recently, Shan et al. (193) discovered that targeting lipid metabolism could sensitize iCCA to chemotherapy. RNA sequencing was performed on PDOs that were sensitive or resistant against gemcitabine. SUMO-specific protease 3 expression was decreased in resistant organoids and it was involved in reprogrammed lipid metabolism in CCA (193). Mitochondrial hyperfusion was also connected to chemotherapy resistance. Treatment with cisplatin increased adaptive mitochondrial hyperfusion, which in turn caused cisplatin resistance. Therefore, targeting mitochondrial morphology could be a possible approach to sensitize CCA against cisplatin (194).

Metabolism studies could also be combined with research on diagnostic techniques using the organoid model. 5-aminolevulinic acid is a non-proteinogenic amino acid and its metabolite accumulates in tumor tissue and can be utilized for photodynamic diagnosis in different cancer types (195). CCA PDOs showed higher photodynamic activity caused by a higher accumulation of 5-aminolevulinic acid metabolite compared organoids derived from cancer-adjacent tissue. Therefore, 5-aminolevulinic acid photodynamic activity could be potentially used in CCA diagnosis (195). These findings shed light on the metabolism of this tumor entity, but further studies are necessary to clarify the specific molecular processes involved in these pathways and to explore the role of other metabolic pathways in the disease. CCA organoids provide a valuable platform for studying the metabolic dynamics of this disease, offering insights into stem cell phenotypes and potential diagnostic applications, while highlighting the need for further research to fully elucidate the underlying molecular mechanisms.

#### 3.2.5 Organoids as model for cholangiocarcinogenesis

Recently, the original theory that iCCA derives from cholangiocytes has been challenged by evidence suggesting a potential hepatocytic origin. Saito et al. (39) used the cholangiocarcinoma model to support the hypothesis that iCCA cells could be derived from hepatocytes. iCCA organoids were cultured under conditions that induced differentiation into cells expressing mature hepatocyte markers, such as albumin and bile acid. Moreover, reseeded iCCA organoids cultivated in a medium that favors hepatocyte-like phenotype, presented less spheres, suggesting a reduction of malignancy through hepatocyte differentiation. Here, it was found that Wnt3a may transform mature hepatocytes into iCCA cells (39). Sun et al. (196) also used the model to investigate the molecular mechanism of the hepatocytic origin of iCCA. The transfection of RAS in reprogrammed human hepatocyte organoids developed typical human iCCA structures after transplantation, which was not the case in RAS transfected reprogrammed hepatocytes in 2D cultures, thus showing the importance of a 3D tumor model (196). To further investigate the origin of CCA, Saborowski et al. genetically modified liver organoids to study cholangiocarcinogenesis. Once transplanted into mice, depending on their genetic profile, they formed tumors with histological characteristics of CCA or hepatocellular carcinoma (197). Using mice transplanted with organoids, Li et al. proved that hepatocellular carcinoma can acquire CCA characteristics during later stages, thus demonstrating that tumors with mixed hepatocellular and CCA features derive from advanced-stage hepatocellular carcinoma (198). Following this discovery, Fan et al. found that Hkdc1 in the organoids was associated with increased metastases in an allograft mouse model (199).

A rare primary liver cancer, known as combined hepatocellular-cholangiocarcinoma, with both hepatocytic and biliary characteristics. Due to its low incidence and biological complexity, this rare malignancy lacks a reliable model. To address this gap, Gao et al. (22) established two combined hepatocellular-cholangiocarcinoma organoids, and these PDOs were characterized through fingerprinting, whole-exome sequencing as well as through analysis of their morphology, growth and histology. The organoids were xenografted into mice and the subcutaneous tumors showcased high levels of similarity to the patients’ tumors (22). In a similar effort, Tang et al. (200) developed a new combined hepatocellular-cholangiocarcinoma cell line and demonstrated its capacity to form tumor spheres under low-attachment conditions. Furthermore, this cell line was successfully used to generate organoids, supporting its utility as a versatile tool for studying this rare tumor entity (200).

Another application of the model for cholangiocarcinogenesis research was to study the two distinct subtypes of iCCA: small duct and large duct. The morphology was similar between the primary tissue and the derived organoids, with the large duct presenting glandular and columnar cells, while the small ducts presented compact, small round cells (23). The advantage of using organoids for subtyping is their ability to facilitate deep analysis despite the often limited availability of tumor tissue (23). Another classification of iCCA was also recently established by Cho et al. (201), who theorized three iCCA groups based on mutations, transcriptomics, proteomics and metabolomics, called “stem-like”, “poorly immunogenic”, and “metabolism”. The “stem-like” CCA is characterized by an overexpression of genes connected to stemness, an increase in the glycolysis pathway and ALDH1A1 overexpression and patients have an intermediate prognosis Therefore, a combination treatment consisting of an ALDH1A1 inhibitor and nab-paclitaxel had a synergic effect in ALDH1A1+ organoids but presented an antagonist effect in the ALDH1A1- organoids (201). These studies focus more on basic research but understanding the origin of a cancer and classifying it thoroughly could be a fundamental step in finding alternative ways to target the malignancy.

#### 3.2.6 Organoids for studying CCA risk factors

The multifaceted organoid research model also allows the study of CCA risk factors. Hepatitis B virus (HBV) is one of the most common risk factors for iCCA. Organoids from HBV+ iCCA patients were cultured and showed positive Hepatitis B surface antigen staining. This led to the conclusion that the virus played a role in the transdifferentiation of hepatocytes into iCCA cells. Since HBV only affects hepatocytes, but not healthy bile duct cells, the surface antigen was declared a “tracer protein” (202) However, even if HBV is a risk factor for CCA, the activation of the immune response caused by the infection could influence the prognosis of this malignancy in a positive way. The immune related gene TNFSF9 was underexpressed in iCCA organoids with HBV infections. Additionally, the growth of non-infected iCCA organoids had a decreased cell viability by inhibiting the expression of TNFSF9 (203).

Primary sclerosing cholangitis (PSC), a chronic inflammation of the bile ducts, is a known risk factor for CCA development. To discover the direct effect of inflammatory cytokines associated with PSC on CCA cell proliferation, Lieshout et al. (204) exposed CCA organoids to various cytokines. IL-17A specifically stimulated cell proliferation, leading to a visible increase in organoid size (204). PSC and CCA organoids were utilized for testing the effect of the JAK inhibitor baricitinib and it inhibited the secretion of different cytokines in the two types of organoids (205). Frank et al. (28) established PDOs from patients with PSC. 5 months after the surgery, one patient was diagnosed with CCA. Therefore, a transcriptomics study was performed, which found an upregulation of cancer-related genes in the organoids of the patient that developed CCA later. This highlights how organoid models could be used for determining CCA prognosis in future applications (28).

Even bacteria have been linked to CCA development. It was concluded that bacteria have an important role in formation of hepatolithiasis, a condition characterized by biliary stones in the intrahepatic bile ducts, which is linked to iCCA progression. First, a 3D spheroid migration assay was performed in an indirect co-culture with Escherichia coli, revealing increased migration of iCCA cells. Then, organoids derived from patients with both iCCA and hepatolithiasis presented greater resistance to gemcitabine (206).Other risk factors for the development of CCA are choledochal cysts, congenital dilations of bile ducts. Using organoids of choledochal cysts, Ye et al. showed that a higher expression of *FGFR2* and *CEBPB* could increase the risk of developing cancer in patients having choledochal cysts (207). Finally, the connection of biliary epithelial injury and cholangiocarcinoma formation was studied. Nakagawa et al. (208) discovered that after biliary epithelial injury the regenerative response mediated through IL-33 increased the development of eCCA. Biliary organoids were created and transplanted into nude mice. The detached, dying biliary epithelial cells released a high quantity of IL-33 (208). The hyperplastic role of IL-33 in the bile ducts was also previously investigated by Razumilava et al. (209). The growth of extrahepatic biliary duct organoids was stimulated by IL-33 and immunohistochemistry demonstrated that IL-33 was overexpressed in CCA tumor tissue. In summary, these studies underline the role of this interleukin in bile duct proliferation and possible involvement in cholangiocarcinogenesis (208, 209).

These findings highlight risk factors associated with carcinogenesis, and further research could explore therapies targeting molecular pathways specific to CCA developed in the presence of other conditions, such as HBV, hepatolithiasis, and choledochal cysts.

#### 3.2.7 Progress in organoid techniques

Organoids are a relative new 3D model, therefore optimizing organoid methodologies and conducting basic research to find novel applications for this model is crucial for its use in tumor research. Roos et al. (33) established cholangiocyte branching organoids to more accurately represent the bile duct architecture, and to better understand the signaling pathways involved in tubular development. Branching CCA organoids better mimicked the primary tumor and presented increased chemoresistance (33). Mi et al. (41) compared undifferentiated organoids and organoids with mature branching phenotype. Branching organoids compared to undifferentiated organoids displayed greater resistance to standard chemotherapeutic treatment, especially to gemcitabine. However, the combination of the treatment with gemcitabine and a Bcl-xl inhibitor was effective, increased apoptosis and changed the morphology of the braching organoids (41).

Conventional hydrogel used for 3D cell culture often contain high concentrations of gelatin, but they can limit cell growth. To address this limitation, an ultra-low-gelatin-content hydrogel was recently developed, that was able to improve both cell spreading and cell growth (210). Furthermore, CCA PDOs have also been cultivated in hydrogels derived from decellularized tumor ECM to more accurately replicate the TME (211). All in all, media composition is of primary importance for proper organoid culture, especially in drug testing (173). Lisky et al. (212) developed a FGFR2 PDO from a PDX. Interestingly, the constitutive activation of FGFR2 allowed the culture of organoids even without growth factors (EGF, FGF10, and HGF) (212).

In order to address one of the main problems of organoids - namely poor reproducibility - Van Tienderen et al. (213) developed an ECM containing microcapsules that allow for scalable and size-controlled growth of CCA PDOs. This encapsulation technique allowed the formation of size-standardized organoids (213). Another frequent challenge is the extended cultivation times required for some PDO lines. Wang et al. found that the addition of lactate to the culture medium increased the growth of hepatopancreatobiliary cancer PDOs, while maintaining their pathological features, genetic profile and chemosensitivity (214). The objective of Roalsø et al. was to discover the feasibility of the establishment of organoids following pancreatectomy. Only one patient in this study presented dCCA and a PDO was established successfully from the resected tissue, later it was cryopreserved for biobanking (215). However, in the realm of PDOs, less invasive methods for obtaining cancer cells from patients compared to the aforementioned surgery, are highly encouraged. Kinoshita et al. established a method to generate organoids from the bile of patients with cholangiocarcinoma, using an endoscopic retrograde cholangiopancreatography (216).

Organoids can also be used to establish mouse models, which can then be utilized in other experiments. Kasuga et al. created a new mouse model through injecting cells with biliary epithelial stem properties derived from KRAS(G12V)-expressing organoids (186). Then, these mouse tumors were compared to human CCA tumors through pixel-level clustering of hematoxylin and eosin-stained slides (217). Finally, CCA organoids were analyzed and characterized for better understanding. Single cell RNA sequencing was used to compare primary tumor to the derived PDO (218) and CCA organoids were imaged to define principles and patterns regarding their morphogenesis (219).

Finally, artificial intelligence is increasingly integrated in oncological research, including its potential application with 3D tumor models. Piansaddhayanon et al. established a deep learning model that can distinguish organoid-derived cancer cells from normal cells from CCA patients (220).

Altogether, there is still much improvement needed in the novel organoid model and this research suggests useful techniques to ameliorate this model and make it more accessible to a wider range of laboratories.

### 3.3 3D co-culture

3D co-culture entails the co-cultivation of more than one cell line with extracellular matrix proteins, e.g. Matrigel, to create a three-dimensional structure. It mainly includes heterotypic spheroids and organoids. Thus, the presence of various cell types and their interactions within an extracellular matrix resembles an *in vivo* environment (221). An example of “simple” co-culture is the indirect co-culture of cells using conditioned medium (222). Okabe et al. (223) performed an human umbilical vein endothelial cells tube formation assay with conditioned medium from tumor stimulated HS cells, and from HS cells and tumor cells individually. The results show that tumor cells could have stimulated HS cells to secrete angiogenic factors, thus emphasizing the importance of stromal cell interactions in tumor angiogenesis (223). Raggi et al. (224) created a CCA sphere conditioned medium to cultivate healthy monocytes, that resulted in high macrophage activation. This highlights the importance of the interactions between cancer cells and cells of the microenvironment (224). However, the TME of CCA is far more complex. Besides immune cells such as macrophages, it includes cancer-associated fibroblasts (CAFs), endothelial cells, bile duct epithelial cells, and extracellular matrix components that all contribute to tumor growth, immune evasion, and therapy resistance (225).

Recent advances have enabled the development of heterotypic spheroids. These mixed-cell spheroids better mimic the cellular heterogeneity and complex interactions within the native TME. The spatial organization within these spheroids —such as the localization of proliferative cancer cells at the periphery and more quiescent cells in the core— also mirrors *in vivo* tumor architecture. By incorporating multiple stromal and immune components, these 3D co-culture systems allow researchers to study intercellular communication, drug penetration, and immunomodulation in a highly controlled yet biologically relevant context (226).

Sueca-Comes et al. (227) investigated the addition of mesenchymal stem cells to CCA spheroids. Spheroids were grown as a monoculture and a as coculture with immortalized mesenchymal stem cells from bone marrow. The presence of mesenchymal cells in the co-culture was able to activate important paracrine signals, thus imitating further the TME (227). Gondaliya et al. (228) created an even more realistic heterotypic spheroid model comprising of cancer cells, HS cells, fibroblasts and endothelial cells. Nanovesicles decorated with epithelial cell adhesion molecule were then used to deliver programmed death-ligand 1 RNA therapeutics to CCA spheroids, successfully reducing programmed death-ligand 1 expression and enhancing immune responses (228). Tian et al. (229) focused specifically on role of peritumoral myofibroblasts on cancer cell growth, a special spheroid-based co-culture system was developed. In this system, 3D tumor spheroids containing only cancer cells, and heterotypic spheroids with both cancer cells and intratumoral myofibroblasts, were placed on a 2D layer of peritumoral myofibroblasts. The co-culture was monitored over an extended period, and it was shown that while peritumoral myofibroblasts initially suppressed tumor growth, they later promoted tumor dissemination. In contrast, intratumoral myofibroblasts had the opposite effect (229). Manzanares et al. (230) also previously performed a 3D co-culture with CCA cells and cancer-associated myofibroblast. The 3D organotypic model mimicked the desmoplasmatic stroma of iCCA tumors, characterized by dense fibrous collagen. Moreover, TGF-β was found to be one of the most fundamental drivers of formation of this desmosplatic stroma in this co-culture (230).

Cancer-associated fibroblasts (CAFs) are gaining increasing amounts of attention in cancer research because of their important role in tumor progression and chemotherapy resistance (231). Different studies focused on creating 3D tumor co-culture models containing CAFs (232–237). Campbell et al. developed an organotypic co-culture model with CCA and α-SMA-positive CAFs that produced a desmoplasmatic environment, similar to the tumor *in vivo* from which the cells derived (232). Liu et al. (233) used a 3D co-culture model of organotypic growth in which cholangiocarcinoma cells were co-cultured with CAFs in a rat type I collagen gel matrix. The novel model was used to show that taurocholate was able to mediate an increase in both the number and size of CCA spheroid/duct-like structures (233). A 3D co-culture of CCA and CAFs or mesenchymal stem cells was more resistant to the combination of gemcitabine and cisplatin, and to the monotreatment with erlotinib compared to the monoculture. However, the majority of co-cultured displayed sensitivity to Afatinib (234). CAFs stimulated the 3D growth of iCCA cells, which could be suppressed by inhibiting the placental growth factor, offering a potential strategy to target the desmoplastic microenvironment of iCCA (235). Moreover, CAFs significantly contributed to resistance and metastasis in 3D models (236). Lasty, the Notch1 inhibitor Crenigacest was able to target the cross-talk between iCCA and CAFs (237).

More complex 3D co-culture approaches combine cancer organoids with cells of the TME. Li et al. (238) created CCA PDOs that contained both epithelial cancer cells and CAFs from the corresponding tumor sample. These PDOs were resistant to bortezomib, while organoids consisting only of cancer cells were sensitive to the drug, highlighting the role of CAFs in chemoresistance (238). Guo et al. (239) created an eCCA organoid co-culture model with macrophages. First, tumor-associated macrophages were induced from monocytes present in patient blood, then they were cultured together with the eCCA patient organoids. Thanks to the presence of tumor-associated macrophages, this model better represented tumor heterogeneity, and the co-culture model also exhibited increased levels of chemotherapy resistance (239). CCA organoids were also co-cultivated with peripheral blood mononuclear cells or T cells. The co-culture system resulted in a lower number of living organoid cells and increased cell death caused by the cultivation with immune cells, highlighting the importance of the immune environment of cancers (240).

The most advanced co-culture models include decellularized tissue. Van Tienderen et al. (29) used decellularized cholangiocarcinoma tumor tissue to cultivate cholangiocarcinoma organoids. The transcriptomics of organoids cultivated in decellularized tumor was more similar to the *in vivo* primary tumor. Additionally, the treatment of this model with chemotherapy exhibited increased resistance, suggesting that the desmoplastic environment might play a role in order to enable chemoresistance (29). CCA organoids in decellularized tissue were also used to study metastatic colonization. First, lung and lymph node tissues were decellularized to create an acellular ECM scaffold, on which CCA organoids were grown. The results suggested that metastatic migration and proliferation depended on both the patient’s tumor and the ECM of the target organ in the lung, while tumor tissue had the greatest influence on growth in lymph nodes (241).

Three-dimensional models that incorporate cells or structures of the microenvironment appear to be fundamental for recapitulating the complexity of cancer in the future, since more and more studies are highlighting the importance of the TME.

### 3.4 Bioprinted 3D model and tumors-on-a-chip

3D bioprinting is a series of techniques that is used to “print” *in vitro* models designed to mimic the *in vivo* microenvironment of the tumor (242), and has also been recently applied to bile duct engineering (243). Tumors-on-a-chip are engineered miniature tumors grown on microfluidic devices and could be defined as one of the most complex applications of 3D bioprinting (244, 245). Microfluidic devices can even allow the long-term live cell imaging (246). Mao et al. (247) bioprinted patient-derived iCCA cells into a grid structure. The characterization of the 3D bioprinted iCCA model revealed a high cell viability, continuous cell proliferation and chemotherapeutic resistance (247). The bioprinting technique was used to study the role of stromal cells in CCA. An extrusion bioprinter platform was used to print CCA cells surrounded by stromal cells like CAFs, tumor-associated endothelial cells or tumor-associated macrophages. Malignancy, invasion, metastasis and stemness were particularly increased in the 3D co-culture of cancer cells with CAFs and cancer cells with tumor-associated macrophages (242). Unfortunately, one of the disadvantages of bioprinting is the high cost of this method. For this reason, Breideband et al. (248) developed an open-source device based on a consumer-grade 3D stereolithography apparatus printer, to make bioprinting more accessible, that was then tested by creating successfully encapsulated CCA organoids (248).

An even more advanced model is the 3D-bioprinted CCA on a chip. Microchannels were constructed on a chip to recreate blood vessels and bile ducts. CCA cells and endothelial cells were seeded in separate microchannels, while hepatocyte-like cells were embedded in a matrix, creating an “*in vitro* liver”. Compared to the 2D monoculture, the 3D tri-culture model was more sensitive to the antitumor prodrug cyclophosphamide, likely due to the presence of neighboring functional hepatocyte cells that could metabolize the prodrug into its active form, thus paving the way for novel drug testing strategies (249). Polidoro et al. (250) created a three-channel microfluidic device to obtain a cholangiocarcinoma-on-a-chip that also included part of the immune microenvironment. CCA cells and CAFs were co-cultured in a main channel, while endothelial cells were cultivated in a lateral channel, and later, T cells were injected into the system. Stimulated T cells had an increased migration from the endothelial channel to the tumor compartment compared to unstimulated T cells (250).

Further applications of these microfluidic devices also include drug testing. Sun et al. (251) discovered TBK1 inhibition as a possible strategy to improve CAR-T therapy. Tumor spheroids were placed in the center gel region of a microfluidic device, while CAR-T cells were placed in the side channels. The inhibition of TKB1 was able to enhance the efficacy of CAR-T cells against CCA spheroids (251). Additionally, Liu et al. (252) developed an advanced microfluidic device that combined chip-technology with PDOs for the testing of anti-cholangiocarcinoma drugs. Organoids cultivated in the microfluidic device were treated with trastuzumab emtansine, the drug was able to reduce the size and number of organoids overall, but the response varied significantly between different PDOs. The testing was performed also on a chip co-culture of CCA PDOs and recellularized liver and kidney, resulting in a low hepatorenal toxicity while remaining effective against CCA (252). Although bioprinting tumors is a complex method still in its early stages, this powerful and advanced model holds significant potential for the future of oncological research.

### 3.5 CAM model in cholangiocarcinoma

In tumor research, the CAM model serves as an intermediate step for the translation of preclinical research into clinical applications. Recently, the CAM model has been utilized as additional valuable research tool for studying CCA (62).

Schmidt et al. (253) determined the antiangiogenic effects and suitability of two phthalocyanines as photosensitizer for PDT of cholangiocarcinoma using the CAM model. For this purpose, the blood vessel formation in the developing CAM after the treatment with the two photosensitizers for PDT was observed. Before PDT treatment, the CAM had a regular vascular network with an intact capillary bed. PDT with the photosensitizer zinc phthalocyanine caused the degeneration of the existing vascular network, resulting in non-perfused areas. The development of novel anticancer compounds with antiangiogenic potency represents a crucial approach for future treatment strategies, as CCA also requires the formation of new blood vessels for its growth (253).

Brun et al. (254) developed a novel lysosomotropic small molecule, GNS561, and evaluated its effect on tumor growth in the CAM model. Lysosomes are important for cancer proliferation including angiogenesis, aggressiveness, metastasis, and also influence signaling, thus targeting lysosomes offers a promising approach for anticancer therapy in iCCA. The CCA cell line HuCCT1 was grafted onto the CAM and the treatment with GNS561 demonstrated a significant reduction in tumor growth. This is an example of how tumors grown on the CAM provide a rapid and cost-effective platform for initial preclinical analysis of compound’s effects (254).

Ament et al. (255) used the CAM model to study the role of post-translational fucosylation in iCCA. It was suggested that fucosylation levels are abnormally high in iCCA tissues. *In vitro* treatment with the fucosylation inhibitor 6AF significantly reduced cell proliferation, and the effect was reversed by adding L-fucose. While 6AF treatment did not notably affect apoptosis *in vitro*, Ament et al. were able to show a reduction of tumor growth and increased apoptosis in the *in vivo* CAM model. An iCCA cell line was pretreated with 6AF individually, and combined with L-fucose. The cells were then grown on the CAM. The tumors treated with 6AF alone presented a decreased volume compared to the control, while the addition of L-fucose increased tumor volume. Moreover, the treatment with 6AF decreased proliferation and increased apoptosis; the supplementation with L-fucose reversed both the anti-proliferative and apoptotic effects. These findings suggest that targeting fucosylation could be a promising therapeutic strategy for iCCA (255).

These findings demonstrate that the *in vivo* CAM model has a lot of potential regarding CCA research. The highly multifaceted CAM model could enable the study of various aspects of CCA and the testing of therapeutics in a more physiological setting, paving the way for personalized medicine.

## 4 Discussion

CCA is the second most common type of liver cancer and has a poor prognosis due to diagnosis at late stages of the disease (1, 2). Preclinical research using 3D models, such as organoids and spheroids, is crucial for gaining a deeper understanding of CCA, tumor behavior, and drug resistance, ultimately supporting the development of personalized therapies.

The multifaceted organoid model has especially attracted a lot of attention in cancer research in recent years (139) and has been widely used to study CCA. One of the most used applications for organoids has been the drug testing realm (141): from discovery of new targets to drug panel screening, from testing of drug combinations to targeted therapy approaches. However, the most impressive niche of this application is personalized treatment. Some examples of the first personalized treatment studies using CCA organoids were cited in this review and had promising results (161–163). Thanks to the ability to create PDOs from patient tumor tissue, the next step, would hopefully be to integrate this model in the tumor board decision-making process.

Beside CCA organoids, spheroids also serve as promising experimental models. Spheroids have been integrated in CCA research mainly through the method called “Spheroid/sphere formation assay”, an *in vitro* technique to study the self-renewal of CSCs (256). CSCs play a fundamental role in CCA and are especially involved in its chemoresistance (257), thus implementing methods such as the spheroid model is of the utmost importance. Moreover, the spheroid model has been particularly useful for the pursuit of new therapeutic targets and drug testing, since they better mimic the TME compared to 2D models and this allows for more faithful results (135, 258).

However, even if spheroids are useful models for studying CCA and are one step ahead compared to classical 2D cell culture (16, 259), they often fail to accurately replicate the native tissue architecture and function of the original tissue (102). In contrast, organoids exhibit a higher and more predictable level of cellular organization and interact more significantly with the extracellular matrix (219). This allows them to preserve most of the histological and malignant characteristics of the original tumor, since factors like the targeted cell population, tissue location and oncogenic complexity highly influence CCA development (24, 208). Nevertheless, creating CCA organoids has been less successful compared to other tumor types and still presents some obstacles (153).

CCA is characterized by a highly desmoplastic stroma, which has a fundamental role in influencing cancer growth and chemoresistance (260). Therefore, the development of cancer models that include tumor stroma component is non-negotiable. 3D co-culture is a vast expression, which includes all 3D models that present more than one cell line (221) (spheroid co-culture, organoid co-culture, decellularized tissue model etc.). All these different models have shed more light on the function of CAFs, tumor-associated macrophages, intratumoral and peritumoral myofibroblasts and HS cells in CCA (223, 229, 233, 239). The final aim of studying the tumor stroma would be to find possible alternative therapeutic targets.

Regarding the most advanced techniques in 3D *in vitro* research, approaches that combine biology and engineering, have created interesting techniques and new models. Current efforts are focused on developing even more complex models like assembloids, which integrate different organoids with each other or fuse organoids with other specialized cell types (261). Thanks to its complexity and inclusion of non-cancer cells of the microenvironment, the new assembloid model has a high potential in CCA studies, both in the basic research field as well as for more accurate drug screenings. The main challenge hereby is to involve co-culturing autologous cell types from the same patient; thus, further studies are essential to develop this approach.

Another example of a novel 3D model is the tumor-on-chip, a tumor in miniature created through a microfluidic device in which cancer cells and stroma cells are seeded (244, 245). The tumor-on-a-chip is probably one of the best *in vitro* models regarding mimicking the TME. Unfortunately, this method is not user-friendly and bioprinting is still a costly technique (58, 59, 245, 248). Through methodologies that could lower some of the costs (248) and practices of standardization, this model, possessing high potential, could become more accessible and thus more integrated in CCA research.

Xenografts typically represent advanced tumor stages and grow rapidly, complicating the study of early-stage CCA. Additionally, different CCA cell lines vary in their ability to form tumors (31). These tumors are implanted in non-physiological sites, rarely metastasize, and may lose the molecular heterogeneity of human CCA (185). This makes it difficult to study interactions between tumor cells, the immune system and microenvironment. Patient-derived xenografts like the CAM model, typically retain the original genetic and epigenetic features. Therefore, the CAM model is promising for predicting therapeutic responses and advancing personalized medicine (262). However, the model is highly sensitive to the external microenvironment and the experiment and observation time is short due to the fast embryo development (62, 263).

Preclinical models are crucial for developing novel clinical strategies for CCA, especially regarding drug discovery (264). Traditional 2D cell cultures, though widely used, have limitations in accurately replicating the original tumor characteristics (9). A key limitation of experimental models in general is their inability to fully capture the complexity of tumor biology and the unique cancer traits of individual patients. For instance, the TME, consisting of a complex interplay between cancerous and non-cancerous cells, creates a desmoplastic environment. Additionally, the considerable heterogeneity within and among tumors is difficult to replicate in models but remains essential for understanding drug resistance and tumor progression (265).

To address these limitations, 3D models like organoids, spheroids and 3D co-culture have been developed, offering a better mimicry of the tumor architecture (9, 10). Unfortunately, the models face some challenges, mainly lack of standardization, reproducibility and high costs (17, 18, 48, 49, 51, 54, 266). Researchers still encounter the dilemma of which model to choose for conducting their research. The simple, standardized and reproducible 2D culture of a commercial cell line in a petri dish is unfortunately far from the pathophysiological conditions of *in vivo* tumors. On the other hand, methods like organoids, which mimic the pathological TME and its heterogeneity, are expensive and not highly reproducible (19). New methodologies are being studied to improve reproducibility, accelerate the growth and lessen the cost of 3D models (213, 214, 248), thus slightly tipping the scale in favor of 3D models. However, this does not mean the extinction of classical 2D culture - it is still and will be a good model for drug screening (267) and for biomarker discovery (268) - but the addition of more variety in models, that will improve research of CCA.

## Conclusion

Disease models should ultimately facilitate the transfer of knowledge from basic laboratory research into clinical applications, enhancing our understanding of the disease and enabling the development of innovative therapies. Since the choice of model is highly dependent on the specific research question, it is strongly recommended to gather results using various models to ensure a comprehensive representation of the tumor. This approach supports the consolidation of scientific data with well-defined minimum criteria before validating these findings through *ex vivo* sample manipulation or clinical trials in patients.

## Supplementary material

Supplementary Table 1 Overview of cited publications regarding CCA organoids, spheroids and 3D co-culture models, describing culture conditions, cells/tissue used for their establishment and methods for characterization of the 3D models.
