# Supplementary material for: Applications of 3D models in cholangiocarcinoma
Source: Front Oncol. 2025 Jul 31;15:1598552. doi: 10.3389/fonc.2025.1598552 (PMC12350147; doi:10.3389/fonc.2025.1598552)
Supplement: Supplementary Table 1 — Overview of cited publications regarding CCA organoids, spheroids and 3D co-culture models, describing culture conditions, cells/tissue used for their establishment and methods for characterization of the 3D models. [file DataSheet1.pdf]

	Reference	DOI	Different types (number)	culture medium composition	culture period	scaffold / matrices	origin of cells	characterization
**Organoids**								
	Koch et al. 2022	10.3390/cells11223613	Icca organoids + intrahepatic cholangyocyte organoids	Liver expansion medium without Wnt and Noggin + 10 µM ROCK inhibitor (Y-27632).	/	Matrigel	ICCA tissue and healthy liver tissue	Xenografting, whole-exome sequencing (Broutier et al. 2017)
	Kaldjob-Heinrich et al. 2025	10.1016/j.gastha. 2024.11.006	1	DMEM/F-12 (GIBCO) supplemented with 1:50 B-27 (GIBCO), 1:100 N-2 (GIBCO), 10 mM nicotinamide (Sigma), 1.25 mM N-acetyl-L-cysteine (Sigma), 10 nM [Leu15]-gastrin (Sigma), 10 µM forskolin (Tocris), 5 µM A83-01 (Tocris), 50 ng/mL EGF (PeproTech), 100 ng/mL FGF10 (PeproTech), 25 ng/ml HGF (PeproTech), 10% RSpo1-conditioned medium, (homemade), and 30% Wnt3a-conditioned medium (homemade).	/	Basement Membrane Extract, Type 2	CCA tissue	Grwoth observation through microscopy, histological staining, tumorigenesis through xenografting, whole exosome sequencing
	Prasopporn et al. 2022	10.3389/fonc. 2022.1021632	2	DMEM/F-12 (GIBCO) supplemented with 1:50 B-27 (GIBCO), 1:100 N-2 (GIBCO), 10 mM nicotinamide (Sigma), 1.25 mM N-acetyl-L-cysteine (Sigma), 10 nM [Leu15]-gastrin (Sigma), 10 µM forskolin (Tocris), 5 µM A83-01 (Tocris), 50 ng/mL EGF (PeproTech), 100 ng/mL FGF10 (PeproTech), 25 ng/ml HGF (PeproTech), 10% RSpo1-conditioned medium, (homemade), and 30% Wnt3a-conditioned medium (homemade).	/	Matrigel	CCA tissue	/
	Suppramote et al. 2022	10.3389/fonc. 2022.877194	2	DMEM/F-12 (GIBCO) supplemented with 1:50 B-27 (GIBCO), 1:100 N-2 (GIBCO), 10 mM nicotinamide (Sigma), 1.25 mM N-acetyl-L-cysteine (Sigma), 10 nM [Leu15]-gastrin (Sigma), 10 µM forskolin (Tocris), 5 µM A83-01 (Tocris), 50 ng/mL EGF (PeproTech), 100 ng/mL FGF10 (PeproTech), 25 ng/ml HGF (PeproTech), 10% RSpo1-conditioned medium, (homemade), and 30% Wnt3a-conditioned medium (homemade).	/	Matrigel	ICCA tissue	/
	Bai et al. 2022	10.3389/fonc. 2022.860339	3	Advanced DMEM/F12 (Gibco) supplemented with 0.1% Bovine Serum Albumin (Sigma) 1×Glutamax, 10 mM HEPES, 1×penicillin/streptomycin, 1×N2 supplement, 1×B27 supplement, 50 ng/mL recombinant human EGF (all from Life Technologies), 1.25 mM N-acetylcysteine, 10 nM [Leu15]-gastrin I human, 10 mM nicotinamide (all from Sigma), 500 ng/ml R-Spondin 1, 100 ng/ml Wnt3a (all from R&D), 5 µM A83-01, 10 µM forskolin (all from Tocris), 100 ng/ml recombinant human FGF10, 25 ng/ml recombinant human HGF, 25 ng/ml Noggin(all from PeproTech). For the first six days of culture, 10 µM ROCK inhibitor Y-27632 (Tocris) was included.	Long-term proliferation	Matrigel or BME-002	CCA tissue	Histological staining
	Bai et al. 2022	10.31083/j.fbl2701018	1	Mouse organoid culture medium (Stemcell, #06030)	/	Matrigel	Primary mouse iCCA cells	/
	Pang et al. 2022	10.1016/j.bioorg. 2022.105679	1	Advanced DMEM/F12 supplemented with 1% penicillin/streptomycin, 10 mM GlutaMAX, 15 mM HEPES, 100 µg/mL Primocin, 1 × B27 supplement (without vitamin A), 1 × N2 supplement, 1 mM N-acetyl-l-cysteine, 10 nM human recombinant (Leu15) -gastrin I, 100 ng/mL recombinant human FGF10, 50 ng/ml human recombinant EGF, 25 ng/ml recombinant human HGF, 100 ng/ml forskolin, 5 µM A8301, 10 µM Y27632 and 3 nM Dexamethasone.	/	Matrigel	Hydrodynamic induced mouse primary intrahepatic cholangiocarcinom a	/
	Cavalloro et al. 2025	10.1016/j.biopha. 2025.118208	2	Advanced DMEM/F-12 (GIBCO) with 1 % penicillin/streptomycin (GIBCO), 1 % Glutamax (GIBCO), 10 mM HEPES (GIBCO) with the addition of B-27 (1:50) (GIBCO), N-2 (1:100) (GIBCO), 10 mM nicotinamide (Sigma-Aldrich), 1.25 mM N-acetyl-L-cysteine (Sigma-Aldrich), 10 µM forskolin (Tocris), 10 µM ROCK inhibitor (Y-27632) (Tocris), 5 µM A83–01 (Sigma-Aldrich), 10 nM [Leu15]-gastrin I (Sigma-Aldrich), 50 ng/mL epidermal growth factor (EGF) (R&D Systems), 25 ng/mL hepatocyte growth factor (HGF) (Homemade), 10 % R-Spondin1 conditioned medium (Sigma-Aldrich) and 30 % Wnt3a conditioned medium with Noggin (Sigma-Aldrich).	/	UltiMatrix Reduced Growth Factor Basement Membrane Extract	iCCA tissue	Growth monitoring through microscopy, histological staining
	Qiao et al. 2023	10.1002/cac2.12452	/	Advanced DMEM/F12 (GIBCO) supplemented with 1% penicillin-streptomycin, 2 mmol/L GlutaMAX, 0.1 mmol/L nonessential amino acids, 10 mmol/L HEPES (GIBCO), 1:50 B27 supplement without vitamin A (GIBCO), 1:100 N2 supplement (GIBCO), 10 mmol/L nicotinamide (Sigma-Aldrich), 1.5 mmol/L N-Acetylcysteine (Sigma-Aldrich), 10 nmol/L recombinant human [Leu15]-Gastrin I (Sigma-Aldrich), 5 µmol/L A8301 (Sigma-Aldrich), 50 ng/mL human recombinant EGF (ThermoFisher Scientific, Waltham, MA, USA), 50 ng/mL human recombinant HGF (ThermoFisher Scientific), 50 ng/mL human recombinant FGF-10 (ThermoFisher Scientific), 10 µmol/L Y-27632(Sigma-Aldrich), and 30% Wnt3a-conditioned medium.	/	Matrigel	CCA tissue	/
	Hosni et al. 2025	10.1158/1535-7163. MCT-24-0972	6 (long term)	Advanced DMEM/F12 supplemented with 1% penicillin/streptomycin, 1% Glutamax, 10-mM HEPES, 1:50 B27 supplement (without vitamin A), 1:100 N2 supplement, 1.25-mM N-acetyl-L-cysteine, 10% (vol/vol) Rspo-1 conditioned medium, 30% (vol/vol) Wnt3a-conditioned medium, 10-mM nicotinamide, 10-nM recombinant human (Leu15)-gastrin I, 50 ng/ml recombinant human EGF, 100 ng/ml recombinant human FGF10, 25 ng/ml recombinant human HGF, 10 µM forskolin, 5 µM A8301, 25 ng/ml Noggin and 10-µM Y27632.	More than 10 passages	Collagen matrix mixture	CCA primary tumors and metastases/relaps es	Growth monitoring through microscopy, histological staining
	Zheng et al. 2021	10.3389/fphar. 2021.723488	/	Huang et al. 2021	/	Matrigel and BME2	CCA tissue	/
	Huang et al. 2024	10.3389/fphar. 2024.1288255.	/	The medium and lysate that were necessary for organoid culture were acquired from ACCURATE biotechnology.	/	Matrigel	CCA tissue	Growth monitoring through microscopy, histological staining

Feng et al. 2024	10.1038/s41419-023-06406-7	/	Complete F medium (1×) For 500 ml, mix 373 ml of complete DMEM, 125 ml of F12 nutrient mix, 0.5 ml of hydrocortisone/ EGF mix, 0.5 ml of insulin, 0.5 ml of amphotericin B, 0.5 ml of gentamicin and 4.3 µl of cholera toxin. Filter-sterilize the solution using a 0.2-µm sterile filter and store it at 4 °Cfor up to 2 weeks (final concentrations: insulin, 5 µg/ml; amphotericin B, 250 ng/ml; gentamicin, 10 mg/ml; cholera toxin, 0.1 nM; EGF, 0.125 ng/ml; hydro-cortisone, 25 ng/ml). After adding the ROCK inhibitor Y-27632 at a final concentration of 10 mM.	/	Matrigel	Mouse tumors	/
Xin et al. 2023	10.1001 /jamanetworkopen. 2023.1476	6	Advanced DMEM/F12 supplemented with 1% penicillin/streptomycin, 1% GlutaMAX-I, 10 mM HEPES, 1:50 B27 supplement, 1:100 N2 supplement, 1.25 mM Nacetyl-L-cysteine, 10 mM nicotinamide, 10 nM Gastin, 50 mg/ml human EGF, 100 ng/ml human FGF10, 25 ng/ml HGF, 10 µm forskolin, 5 µm A8301, 10 µm Y27632, and 3 nM dexamethasone.	/	Matrigel	ICCA tissue	/
Wang et al. 2021	10.1186/s12935-021-02219-w	1	Dulbecco's modified Eagle medium/F12 (Gibco, CS, USA) supplemented with penicillin/streptomycin (1×, ThermoFisher, MA, USA), Glutamax (1×; ThermoFisher), B27 supplement (1×; Gibco, CS, USA), N2 supplement (1×; Gibco, CS, USA), HEPES (10 mM, ThermoFisher, MA, USA), gastrin (10 nM; Sigma Aldrich, MO, USA), A83-01 (5 µM; Tocris, Bristol, UK), Y-27,632 (10 µM; Tocris, Bristol, UK), recombinant human epidermal growth factor (50 ng/mL; PeproTech, NJ, USA), recombinant human fibroblast growth factor 10 (100 ng/mL; PeproTech), recombinant human R-Spondin1 (500 ng/mL; PeproTech,), recombinant human Noggin (100 ng/mL; PeproTech), and Afamin/Wnt3a CM (10% v/v; MBL Life Science, TKY, Japan).	2 weeks or or when the diameters of most PDO had reached 150 µm	Growth factor reduced Matrigel	ECCA tissue	Histological staining, whole-exome sequencing
Saito et al. 2019	10.1016/j.celrep. 2019.03.088	1 xenograft organoid + 2 primary iCCA organoids + 2 non-cancer bile duct organoids	Advanced DMEM/F12 (Thermo Fisher Scientific) supplemented with Glutamax, 10 mM HEPES, penicillin/streptomycin, 1x N2 supplement, 1x B27 supplement, 50 ng/mL EGF (all from Thermo Fisher Scientific), 1.25 mM N-acetylcysteine, 10 nM gastrin, 10 mM nicotinamide (all from Sigma-Aldrich), Rspo1 (10% conditioned medium from Rspo1-producing cell line), 5 µM A83-01 (Tocris), 10 µM forskolin (Tocris) and 10 µM Y-27632 (WAKO).	More than a 1 year (cancer organoids), till passage 15 (non-cancer organoids)	Matrigel	Xenograft tissue from iCCA patient, human iCCA tissue, non-cancer bile duct tissue	Histological staining, microarray analysis, differential expression analysis, whole-exome sequencing
Li et al. 2019	10.1172/jci.insight. 121490	/	Advanced DMEM/F12 supplemented with 1× HEPES, 1× GlutaMax (Invitrogen), 1× primocin (InvivoGen), 1× B27 Supplement (Gibco), 1× N-2 Supplement (Gibco), 30% Wnt-conditioned medium, 20% Rspondin-conditioned medium, 0.5 µM A83-01 (Tocris), 0.05 µg/ml EGF (Sigma-Aldrich), 0.1 µg/ml FGF-10, 0.03 µg/ml HGF (Peprotech), 12.5 µM FSK (LC Laboratories), 0.01 µM gastrin (Sigma-Aldrich), 0.1 µg/ml noggin (Peprotech), 1.25 mM N-acetylcysteine (Sigma-Aldrich), and 12.5 mM nicotinamide (Sigma-Aldrich). For initial establishment of the PDOs, we used 50% (vol/vol) Wnt-conditioned medium and 30% (vol/vol) Rspondin-conditioned medium in the first week, 10 µM Y27632 (LC Laboratories) was added at the PDO establishment and passage step.	/	Growth factor reduced Matrigel	ICCA tissue	Grrowth monitoring through microscopy, histological staining
Lieshout et al. 2022	10.1186/s10020-022-00498-1	3 CCA organoids, 2 cholagiocyte (tumor adjacent) organoids, 3 healthy cholangyocyte organoids	Advanced DMEM/F12 supplemented with 1% penicillin/streptomycin, 1% Glutamax, 10-mM HEPES, 1:50 B27 supplement (without vitamin A), 1:100 N2 supplement, 1.25-mM N-acetyl-L-cysteine, 10% (vol/vol) Rspo-1 conditioned medium, 30% (vol/vol) Wnt3a-conditioned medium, 10-mM nicotinamide, 10-nM recombinant human (Leu15)-gastrin I, 50 ng/ml recombinant human EGF, 100 ng/ml recombinant human FGF10, 25 ng/ml recombinant human HGF, 10 µM forskolin, 5 µM A8301, 25 ng/ml Noggin and 10-µM Y27632.	/	Matrigel/BME	CCA tissue, adjacent non-tumorous tissue, healthy donor liver tissue	Tumorigenicity through xenografting, targeted NGS
Zhao et al. 2021	10.7150/ijbs.67379	/	Advanced DMEM/F-12 supplemented with 1% penicillin/streptomycin, 1% GlutaMAX, 10 mM HEPES, 2% B-27, 1% N-2, 10 mM nicotinamide, 1.25 mM N-acetyl-L-cysteine, 10 nM [Leu15]-gastrin, 10 mM forskolin, 5 mM A83-01, 50 ng/mL EGF, 25 ng/ml HGF, 0.1% Plasmocin, 10% RSpo1-conditioned medium (homemade), and 30% Wnt3a-conditioned medium (homemade).	/	Matrigel	Cancer cells from mice tumors	/
Broutier et al. 2017	10.1038/nm.4438.	2 combined HCC/CCA organoids + 3 CCA organoids + healthy organoids	Classical isolation medium or tumoroid-specific isolation medium (classical isolation medium without R-spondin-1, Noggin and Wnt3a, but supplemented with dexamethasone and Rho-kinase inhibitor), then all cultures cultivated in "human healthy liver-derived organoids expansion medium".	1 year circa	Matrigel/BME	CCA and combined HCC/CCA tissue, healthy liver tissue	Histological staining, RNA sequencing, whole-exome sequencing, metastatic potential when transplanted
Maier et al. 2021	10.3390/ijms22168675	29 cancer organoids (2 organoids for in-depth characterization) + 1 benign organoid	50 % Wnt3a, 24.6% AdDMEM/F12 (Invitrogen), 10% R-spondin (R&D Systems), 10 %Noggin (Thermo Fisher Scientific), 2% B27-Supplement 50x (Life Technologies), 1% Nicotinamide (10 mM) and 1% Insulin-Transferrin-Selenium (ITS) 100x (both from Sigma Aldrich), 0.4% A83.01 (2µM) (Tocris), 0.2% N-Acetyl-L-Cysteine (1 mM) (Sigma Aldrich), 0.2% FGF10(200 ng/ml) (PeproTech), 0.2% Primocin (Invivogen), 0.1% Y-27632 (Sigma Aldrich), 0.1%EGF (1:10, PeproTech), 0.1% Gastrin 1 µM (Sigma Aldrich) and 0.1% Forskolin (Tocris).	1 - 103.3 weeks	Matrigel	CCA tissue and non-cancer bile duct tissue	Histological staining, tumorigenicity through transplantation, RNA sequencing, transcriptomic analysis

Wang et al. 2021	10.3390 /cancers13051179	1	Advanced Dulbecco's modified Eagle medium/F12 (Gibco, Carlsbad, CA, USA) supplemented with 1× penicillin/streptomycin (ThermoFisher, Waltham, MA, USA), 1× Glutamax (ThermoFisher, Waltham, MA, USA), 10 mM N-2-Hydroxyethylpiperazine-N-2-Ethane Sulfonic Acid (ThermoFisher, Waltham, MA, USA), 1× B27 supplement (Gibco, Carlsbad, CA, USA), 1× N2 supplement (Gibco, Carlsbad, CA, USA), 10 mM nicotinamide (Sigma, St. Louis, MO, USA), 1.25 mM N-acetyl-L-cysteine (Sigma, St. Louis, MO, USA), 10 nM gastrin (Sigma, St. Louis, MO, USA), 5 μM A83-01 (Tocris, Bristol, UK), 50 ng/mL recombinant human epidermal growth factor (PeproTech, Rocky Hill, NJ, USA), 100 ng/mL recombinant human fibroblast growth factor 10 (PeproTech, Rocky Hill, NJ, USA), 10 μM Y-27632 (Tocris, Bristol, UK), 500 ng/mL recombinant human R-Spondin1 (PeproTech, Rocky Hill, NJ, USA), 10% Afamin/Wnt3a CM (MBL Life Science, Kyoto, Japan), and 100 ng/mL recombinant human Noggin (PeproTech, Rocky Hill, NJ, USA).	3 weeks ?	Growth factor reduced Matrigel	ICCA tissue	Histological staining, whole-exome sequencing
Ren et al. 2023	10.1016/j.xcrm. 2023.101277	57	Advanced DMEM/F12 supplemented with 1% Pen/Strep, 1% glutamax (Gibco), 10-mM HEPES (Gibco), 1:50 B27 supplement without vitamin A (Thermo Fisher Scientific, Massachusetts, USA), 1:100 N2 supplement (Thermo Fisher Scientific), 1.25-mM N-acetyl-L-cysteine (Sigma), 10-mM nicotinamide (Sigma), 10-nM recombinant human (Leu15)-gastrin I (Genscript, Nanjing, China), 50 ng/mL recombinant human EGF (Gibco), 100 ng/mL recombinant human FGF10 (Novoprotein, Suzhou, China), 25 ng/mL recombinant human HGF (Novoprotein), 10 μM forskolin (Sigma), 5 μM A83-01 (TOCRIS, Bristol, England) and 10 μM Y27632(Selleck, Houston, USA).	Long- and short-termed growth	Matrigel	ICCA and eCCA tumor tissue	Histological staining, RNA sequencing, protein expression
He et al. 2024	10.4251/wjgo.v16.i10. 4274	1	Culture medium (Kingbio Medical Co., Ltd., Chongqing, China)	/	Matrigel	PCCA tissue	Growth monitoring through microscopy
Fujiwara et al. 2019	10.1038/s41598-019-55211-w	/	Advanced DMEM/F12 (Invitrogen) supplemented with B27 (Invitrogen), N2 (InvivoGen, San Diego, CA), 1.25 μM N-acetylcysteine (Wako, Osaka, Japan), 50 ng/mL EGF (Wako), 100 ng/mL FGF10 (Wako), 50 ng/mL HGF (R&D Systems, Minneapolis, MN), 10% RSPO1-conditioned medium, 10 nM gastrin (Sigma-Aldrich), and 10 mM nicotinamide (Sigma-Aldrich). For the first 4 d after seeding, the cells were also supplemented with 100 ng/mL Noggin (PeproTech, Rocky Hill, NJ) and 10% Wnt3a-conditioned medium.	/	Matrigel	Murine liver tissue	Histological staining and?
Luk et al. 2024	10.1126/scitranslmed. adj7685	3	AdDMEM/F12 medium supplemented with HEPES [1×, Invitrogen], Glutamax [1×, Invitrogen], penicillin/streptomycin [1×, Invitrogen], B27 [1×, Invitrogen], Primocin [1 mg/ml, InvivoGen], N-acetyl-L-cysteine [1 mM, Sigma], Wnt3a-conditioned medium [50% v/v], RSPO1-conditioned medium [10% v/v, Calvin Kuo], Noggin-conditioned medium [10% v/v] or recombinant protein [0.1 μg/ml, Peprotech], epidermal growth factor [EGF, 50 ng/ml, Peprotech], Gastrin [10 nM, Sigma], fibroblast growth factor 10 [FGF10, 100 ng/ml, Preprotech], Nicotinamide [10 mM, Sigma], and A83-01 [0.5 μM, Tocris]).	/	Matrigel	ICCA tissue	/
Cristinziano et al. 2021	10.1016/j.jhep. 2021.02.032.	5	The expansion medium is composed by Advanced DMEM/F12 supplemented with 1% (vol/vol) penicillin/streptomycin, 1% (vol/vol) Glutamax, 10 mM HEPES, 1:50 B27 supplement (without vitamin A), 1 mM N-acetyl-l-cysteine, 5% (vol/vol) Rspo-1 conditioned medium, 10 mM nicotinamide, 10 nM recombinant human (Leu15)-gastrin I, 50 ng/ml recombinant mouse EGF, 100 ng/ml recombinant human FGF10, 50 ng/ml recombinant human HGF. The deprived medium (DM) is composed by Advanced DMEM/F12 supplemented with 1% (vol/vol) penicillin/streptomycin, 1% (vol/vol) Glutamax, 10 mM HEPES, B27 supplement at 1:50 dilution (without vitamin A), 1 mM N-acetyl-l-cysteine, 10 mM nicotinamide, and 10 nM recombinant human (Leu15)-gastrin I).	/	Growth factor reduced Matrigel/Matrigel	C57BL/6J mice livers	Growth monitoring through microscopy, RT-PCR, histological staining, tumorigenicity through tranplantation
Hogenson et al. 2022	10.1172/jci.insight. 158060.	2	DMEM plus GlutaMax (Gibco, 10564-011), 0.1% Penicillin-Streptomycin-Amphotericin B Solution, 0.25 μg/mL hydrocortisone (Sigma-Aldrich, H0888), 1% B27 (Gibco, 12587-010), 50 μg/mL L-ascorbic acid (Sigma-Aldrich, A92902), 20 μg/mL insulin (Sigma-Aldrich, I9278), 100 ng/mL FGF2 (R&D Systems, 233-FB), and 100 nM all-trans retinoic acid (Sigma-Aldrich, R2625).	/	Growth factor reduced matrigel	CCA tissue	Histological staining, sequencing
Cigliano et al. 2024	10.1186/s13046-024-03177-7	/	HepatiCult Organoid Kit (Human) (STEMCELL Technologies)	/	Growth factor reduced Matrigel/Matrigel	ICCA tissue	/
Elurbide et al. 2024	10.1136/gutjnl-2024-332998	/	Advanced DMEM/F12 supplemented with 1% penicillin/streptomycin, 1% Glutamax, 10-mM HEPES, 1:50 B27 supplement (without vitamin A), 1:100 N2 supplement, 1.25-mM N-acetyl-L-cysteine, 10% (vol/vol) Rspo-1 conditioned medium, 30% (vol/vol) Wnt3a-conditioned medium, 10-mM nicotinamide, 10-nM recombinant human (Leu15)-gastrin I, 50 ng/ml recombinant human EGF, 100 ng/ml recombinant human FGF10, 25 ng/ml recombinant human HGF, 10 μM forskolin, 5 μM A8301, 25 ng/ml Noggin and 10-μM Y27632.	/	? Collagen matrix mixture	CCA tissue	Histological staining, expression profile, genomic landscape and neoplastic behaviour of the original tumours
Fu et al. 2024	10.1016/j.apsb. 2024.06.028	3	Complete CCA PDO media	Up to 10 passages	Matrigel	CCA tissue	/
Pan et al. 2024	10.1158/0008-5472. CAN-24-0088	/	Medium with growth factors	/	Basement matrix	CCA tissue	/
Li et al. 2025	10.1097/HEP. 0000000000001423	Tecnical informations regarding organoids not present in the main text						

Chen et al. 2025	10.1158/0008-5472.CAN-24-2097	4	Advanced DMEM/F12 (Gibco) supplemented with 1% penicillin–streptomycin (Gibco), 100 µg/mL Primocin (InvivoGen), 10 mmol/L HEPES (Gibco), 1% GlutaMAX (Gibco), 1:100 N2 supplement (Gibco), 1:50 B27 supplement (Gibco), 500 ng/mL R-spondin 1 (ABclonal), 25 ng/mL Noggin (Sino Biological Inc.), 1.25 mmol/L N-acetylcysteine (Sigma), 10 nmol/L Gastrin (MedChemExpress), 10 mmol/L nicotinamide (Sigma), 50 ng/mL recombinant human EGF (ABclonal), 100 ng/mL recombinant human FGF10 (ABclonal), 10 µmol/L forskolin (MedChemExpress), 10 µmol/L Y27632 (Selleck Chemicals), and 5 µmol/L A83-01 (Selleck Chemicals).	/	Matrigel	ICCA	RT-qPCR
Lampis et al. 2018	10.1053/j.gastro.2017.10.043	1	Advanced Dulbecco's modified Eagle medium/F12, supplemented with 1x B27 additive and 1x N2 additive (Thermo Fisher Scientific), 0.01% bovine serum albumin, 2 mmol/L L-glutamine, 100 units/mL penicillin-streptomycin, and containing the following additives: epidermal growth factor, noggin, R-spondin 1, gastrin, fibroblast growth factor-10, fibroblast growth factor F-basic, Wnt-3A, prostaglandin E2, Y-27632, nicotinamide, A83-01, SB202190, and hepatocytes growth factor (Pepro-Tech, London, UK).	Used for in vitro application and manipulation within 6–8 weeks from establishment.	Growth factor reduced matrigel	ICCA tissue	Histological staining, DNA sequencing, NanoString Analysis
Zhang et al. 2024	10.1186/s12964-024-01500-5	/	DMEM/F12 supplemented with B27, N2, 1.25 mM N-acetylcysteine, 10 mM HEPES, 10 mM nicotinamide, 10 nM gastrin-I, 10 µM Forskolin, 5 µM A83–01 (TGFβ inhibitor), 3 nM dexamethasone, 100 ng/ml FGF10, 50 ng/ml EGF, 25 ng/ml HGF.	/	Matrigel	CCA tissue	/
Artegiani et al. 2019	10.1016/j.stem.2019.04.017.	4	Advanced DMEM/F12 (Invitrogen) supplemented with: 1X B27 (both from GIBCO), 1.25 mM N-Acetylcysteine (Sigma), 10 nM gastrin (Sigma), 50 ng/ml EGF (Peprotech), 10% RSPO1 conditioned media (homemade), 100 ng/ml FGF10 (Peprotech), 25 ng/ml HGF (Peprotech), 10 mM Nicotinamide (Sigma), 5 µM A83.01 (Tocris), 10 µM FSK (Tocris), 25 ng/ml Noggin (Peprotech), 30% Wnt conditioned media (homemade), and 10 uM Y27632 (Sigma Aldrich). After 3-4 days, the isolation medium was changed into a medium without Noggin, Wnt conditioned media, Y27632.	At least 12 months	BME (Amsbio)	Liver tissue	Genotyping through DNA sequencing, histological staining, transmission electron microscope imaging, time-lapse live-cell imaging, mass spectrometry, RNA sequencing, western blot, ChIP, ATAC sequencing, FACS
Kasuga et al. 2021	10.1111/cas.14703.	6 primary organoids + secondary organoids	DMEM‐F12 supplemented with recombinant human hepatocyte growth factor (50 ng/mL, Biolegend), recombinant human epidermal growth factor (50 ng/mL; Pepro Tech), recombinant human fibroblast growth factor 10 (100 ng/mL, Biolegend), nicotinamide (10 mmol/L, Sigma‐Aldrich), recombinant human R‐spondin–1 (0.5 µg/mL, Miltenyi Biotec), B27 supplement without vitamin A (Life Technologies), and Y‐27632 (10 µmol/L).	/	Matrigel	Mouse biliary epithelial cells and tumors from transplanted mice	Histological staining, flow cytometric analysis
Guan et al. 2025	10.1002/advs.202409173	2	/	/	Matrigel	ICCA PDX mouse tissue	Tumorigenicity through transplantation
Conboy et al. 2023	10.1016/j.jhep.2022.09.014	8	DMEM/F12, 1% Glutamax, HEPES, Penicillin-Streptomycin, Noggin, R-Spondin, EGF, HGF, Gastrin, B27, NAC, Y2763, Wnt3A, FGF10, A83-01, Nicotinamide, N2, Forskolin	Experiments within 10 passages from generation	Matrigel	CCA tissue	Histological staining
Conti Nibali et al. 2024	10.1016/j.isci.2024.109853	5 tumor and 5 non-tumor organoids	Advanced DMEM/F-12 (GIBCO) with 1% penicillin/streptomycin (GIBCO), 1% Glutamax (GIBCO), 10 mM HEPES (GIBCO) with the addition of B-27 (1:50) (GIBCO), N-2 (1:100) (GIBCO), 10 mM nicotinamide (Sigma-Aldrich), 1.25 mM N-acetyl-L-cysteine (Sigma-Aldrich), 10 µM forskolin (Tocris), 10 µM Y-27632 dihydrochloride (ROCK inhibitor) (Tocris), 5 µM A83-01 (Sigma-Aldrich), 10 nM [Leu15]-gastrin I (Sigma-Aldrich), 50 ng/ml epidermal growth factor (EGF) (R&D Systems), 25 ng/ml hepatocyte growth factor/scatter factor (HGF/SF) (Homemade), 10% R-Spondin1 conditioned medium (Sigma-Aldrich) and 30% Wnt3a conditioned medium with Noggin (Sigma-Aldrich).	Long-term expansion for tumor organoids, non-tumor organoid (only a few months)	UltiMatrix Reduced Growth Factor Basement Membrane Extract	ICCA and non-tumor liver tissue	Histological staining
Huang et al. 2024	10.1038/s41419-024-06775-7	2	Advanced DMEM/F12 medium, HEPES, Glutamax, Penicillin, Streptomycin, B27, n-Acetylcysteine, EGF, R-spondin1, Noggin, Wnt, FGF10, Gastrin, TGF-inhibitor, and RHOK-inhibitor.	/	Growth factor reduced Matrigel	ICCA tissue	Histological staining
Yoshikawa et al. 2019	10.3390/cancers11121993	2	The glucose-containing medium was Advanced DMEM/F12 (Thermo Fisher Scientific, Waltham, MA, USA). The glucose-free medium was SILAC Advanced DMEM/F12 Flex Media, without glucose or phenol red (Thermo Fisher Scientific). They were both supplemented with 1× Glutamax, 10 mM HEPES, 1× penicillin/streptomycin, 1× N2 supplement, 1× B27 supplement, 50 ng/mL EGF (all from Thermo Fisher Scientific), 10 nM gastrin, 10 mM nicotinamide (all from Sigma-Aldrich, St. Louis, MO, USA) and Rspo1 (10% conditioned medium from Rspo1-producing cell lines).	/	Matrigel	ICCA tissue	Growth monitoring through microscopy, qRT-PCR
Zhang et al. 2021	10.1111/liv.14692	3 CCA organoids + 3 healthy liver organoids	Advanced/F12 DMEM with 1% penicillin/streptomycin, 1% L‐Glutamine and 10 mM HEPES, supplemented with 1:50 B27 supplement (without vitamin A), 1:100 N2 supplement, 1 mM N‐acetylcysteine, 10% (vol/vol) R‐spondin 1 (conditioned medium), 10 mM nicotinamide, 10 nM recombinant human [Leu15]‐gastrin I, 50 ng/mL recombinant human EGF, 100 ng/mL recombinant human FGF10, 25 ng/mL recombinant human HGF, 10 µM Forskolin and 5 µM A83‐01. L‐Arginine was firstly added to "Advanced DMEM/F‐12 without L‐Lysine, L‐Arginine and no‐phenol red" medium, and then different concentrations of L‐Lysine were added.	/	Matrigel	CCA tissue and intrahepatic biliary epithelial progenitor cells	mRNA expression

Li et al. 2020	10.3390/cells9010121	2 CCA organoids + 2 normal organoids	Liver expansion medium without Wnt and Noggin + 10 μM ROCK inhibitor (Y-27632); Advanced DMEM/F12 supplemented with 1% penicillin/streptomycin, 1% Glutamax, 10-mM HEPES, 1:50 B27 supplement (without vitamin A), 1:100 N2 supplement, 1.25-mM N-acetyl-L-cysteine, 10% (vol/vol) Rspo-1 conditioned medium, 30% (vol/vol) Wnt3a-conditioned medium, 10-mM nicotinamide, 10-nM recombinant human (Leu15)-gastrin I, 50 ng/ml recombinant human EGF, 100 ng/ml recombinant human FGF10, 25 ng/ml recombinant human HGF, 10 μM forskolin, 5 μM A8301, 25 ng/ml Noggin and 10-μM Y27632.	/	Broutier et al. 2016; Broutier et al. 2017; Vincent et al. 2019	CCA tissue, tissue adjacent to tumor, donor liver	Histological staining
Shan et al. 2025	10.1016/j.canlet. 2025.217770	18	Complete medium	/	Matrigel	ICCA tissue	Histological staining, RNA sequencing
Lv et al. 2025	10.1016/j.jhep. 2025.03.028.	/	Medium with EGF (PeproTech, Cat#AF-100-15-500), FGF10 (PeproTech, Cat#100-26-25), HGF (PeproTech, Cat#100-39-10), Noggin (Sino Biological, Cat#50688-M02H), R-spondin 1 (Sino Biological, Cat#11083-HNAS), n-Acetylcysteine (Sigma, Cat#A9165), Gastrin (Sigma, Cat#G9145), Nicotinamide (Sigma, Cat#N0636), A83-01 (MCE, Cat#HY-10432), Forskolin (MCE, Cat#HY-15371), Y27632 (Selleck, Cat#S1049), N-2 Supplement (100X) (Gibco, Cat#17502048), B-27 Supplement (50X) (Gibco, Cat#17504044).	/	Matrigel	CCA tissue	/
Fujiwara et al. 2020	10.1016/j.suronc. 2020.10.011	4 CCA organoids + 2 tumor-adjacent organoids	Advanced DMEM/F12 (Invitrogen) supplemented with B27 (Invitrogen), N2 (InvivoGen, San Diego, CA), 1.25 μM N-acetylcysteine (Wako, Osaka, Japan), 10 μM Y-27632 (Wako), 50 ng/mL EGF (Wako), 100 ng/mL FGF10 (Wako), 50 ng/mL HGF (R&D Systems, Minneapolis, MN), 10% RSPO1-conditioned medium, 10 nM gastrin (Sigma-Aldrich), and 10 mM nicotinamide (Sigma-Aldrich). 100 ng/mL Noggin (PeproTech, Rocky Hill, NJ) and 10% Wnt3a-conditioned medium were supplemented for the first 4 days after seeding.	2-3 months	Matrigel	CCA tissue and adjacent tissue	Histological staining, RT-qPCR, tumorigenicity through xenografting
Saito et al. 2018	10.1038/s41598-018-21121-6	/	The expansion medium (EM) was composed of Advanced DMEM/F12 (Thermo Fisher Scientific) supplemented with Glutamax, 10 mM HEPES, penicillin/streptomycin, 1 × N2 supplement, 1 × B27 supplement, 50 ng/mL EGF (all from Thermo Fisher Scientific), 1.25 mM N-acetylcysteine, 50 nM gastrin, 10 mM nicotinamide (all from Sigma-Aldrich) and R-spondin 1 (10% conditioned medium from R-spondin 1-producing cell lines). For culture of organoids derived from IHCC patients #2 and #3, 5 μM A83-01 (Tocris) and 10 μM forskolin (Tocris) were added.	Over a year	Matrigel	ICCA tissue and xenograft tissue of iCCA	Growth monitoring through microscopy, histological staining, western blot, tumorigenicity through transplantation
Sun et al. 2019	10.1038/s41556-019-0359-5	/	DMEM/F12 with Galactose, Ornithine, Proline, Nicotinamide, $ZnCl_2$, $ZnSO_4·7H_2O$, $CuSO_4·5H_2O$, $MnSO_4$, 100x ITS, TGFα (40μg/mL), EGF(40μg/mL), Dex(100mM), Penicillin streptomycin combination	/	Matrigel	Reprogrammed human hepatocytes	Histological staining, transmission electron microscopy, expression profile analysis, qPCR
Saborowski et al. 2019	10.1002/hep4.1312.	/	Liver expansion medium without Wnt and Noggin + 10 μM ROCK inhibitor (Y-27632).	/	Growth factor reduced matrigel	Murine livers	Histological staining, flow cytometry, tumorigenicity through xenografting
Li et al. 2018	10.1016/j.ajpath. 2017.11.013	5	Advanced Dulbecco's Modified Eagle Medium: Nutrient Mixture F-12 medium (Thermo Fisher Scientific) supplemented with 2% B27, 1% N2 (both from Thermo Fisher Scientific), 10% Pen-Strep, and 10% l-glutamine, and mixed with 50% Wnt3A conditioned media from L Wnt-3A cells (CRL-2647; ATCC, Manassas, VA) supplemented with 1.25 mmol/L N-acetyl cysteine (Sigma-Aldrich), 10 nmol/L Gastrin (Sigma-Aldrich), and growth factors, including 50 ng/mL epidermal growth factor (PeproTech, Rocky Hill, NJ), 1 μg/mL R-spondin1, 100 ng/mL Noggin, 100 ng/mL fibroblast growth factor (FGF)-10, 50 ng/mL hepatocyte growth factor (all from R&D Systems, Minneapolis, MN), and 10 mmol/L Nicotinamide (Sigma-Aldrich).	/	Growth factor reduced matrigel/ matrigel	Murine liver tumor tissue	RNA sequencing, tumorigenicity through transplantation
Fan et al. 2024	10.1097/HEP. 0000000000001085	/	Advanced Dulbecco's Modified Eagle Medium: Nutrient Mixture F-12 medium (Thermo Fisher Scientific) supplemented with 2% B27, 1% N2 (both from Thermo Fisher Scientific), 10% Pen-Strep, and 10% l-glutamine, and mixed with 50% Wnt3A conditioned media from L Wnt-3A cells (CRL-2647; ATCC, Manassas, VA) supplemented with 1.25 mmol/L N-acetyl cysteine (Sigma-Aldrich), 10 nmol/L Gastrin (Sigma-Aldrich), and growth factors, including 50 ng/mL epidermal growth factor (PeproTech, Rocky Hill, NJ), 1 μg/mL R-spondin1, 100 ng/mL Noggin, 100 ng/mL fibroblast growth factor (FGF)-10, 50 ng/mL hepatocyte growth factor (all from R&D Systems, Minneapolis, MN), and 10 mmol/L Nicotinamide (Sigma-Aldrich).	/	Growth factor reduced matrigel/ matrigel	Liver cancer mouse model tissue	RNA sequencing, tumorigenicity through transplantation (Organoid lines previously established and described in Li et al. 2018). Gene expression analysis, western blot.
Gao et al. 2024	10.1007/s13577-024-01148-w	2 cancer organoids + 2 cancer-adjacent organoids	Advanced DMEM/F12 medium supplemented with 30% (vol/vol) Wnt-3A conditioned medium (CM), 10% (vol/vol) Rspo-1 CM, 1% penicillin/streptomycin (Invitrogen, Carlsbad, CA, USA), 1% Glutamax (Invitrogen), 10 mM HEPES (Sigma-Aldrich, St. Louis, MO, USA), 1:50 B27 (Thermo Fisher Scientific), 1:100 N2 supplement (Thermo Fisher Scientific), 2.0 μM A8301 (R&D), 1.25 mM N-acetyl-l-cysteine (Sigma-Aldrich), 10 μM forskolin (R&D), 10 mM nicotinamide (Merck KGaA, Darmstadt, Germany), 10 μM rock inhibitor Y27632 (R&D), 10 nM recombinant human (Leu15)-gastrin I (Thermo Fisher Scientific), 50 ng/mL recombinant human EGF (R&D), 100 ng/mL recombinant human FGF10 (R&D), 25 ng/mL recombinant human HGF (R&D), and 50 ng/mL recombinant human noggin (R&D).	More than year (over 50 generations) for the cancer organoids	Type 2 Basement Membrane Extract	Combined hepatocellular CCA tissue and adjacent tissue	Anchorage-independent growth assay, whole-exome sequencing, STR analysis, histological staining, tumorigenicity through transplantation
Tang et al. 2025	10.1186/s12885-025-13876-9.	1	RPMI-1640 [Gibco] + 10% FBS [VivaCell] + 1% penicillin–streptomycin [VivaCell]	/	Matrigel	Combined hepatocellular CCA cell line	Growth monitoring through microscopy

Lee et al. 2023	10.1038/s41467-023-35896-4	16	Advanced D-MEM/F-12 500ML (cat no. 12634010), B-27 supplement without A (50X) 10 ML (cat no. 12587010), N2 SUPPLEMENT (100X) 5ML (cat no. 17502048), N-acetyl-L-cysteine (cat no. A7250), R-spondin conditioned medium, Nicotinamide (cat no. N0636), [Leu15]-gastrin I human (cat no. G9145), Recombinant human EGF (cat no. AF-100-15), Recombinant human FGF10 (cat no. 100-26), rhHGF (cat no. 100-39), Forskolin (cat no. 1099), A83-01 (TGFβ inhibitor) (cat no. 2939), primocin (cat no. ant-pm-1).	/	Matrigel	ICCA tissue	Histological staining, Karyotype, Growth kinetics, Somatic mutation
Cho et al. 2023	10.1053/j.gastro. 2023.02.045.	2	Advanced DMEM/F12 medium (Invitrogen, USA) containing B27 (Invitrogen, USA), 1.25 mM N-acetylcysteine (Sigma-Aldrich), 50 ng/mL EGF (PeproTech, Cranbury, NJ, USA), 10% R-spondin 1 conditioned media (obtained from HA-R-spondin 1-Fc 293T cell), 10 nM gastrin (Sigma-Aldrich), 10 mM nicotinamide (Sigma-Aldrich), 5 μM A83-01 (Tocris, Bristol, UK), 10 μM Forskolin (Tocris, Bristol, UK), 10 μM Y-27632 (Sigma-Aldrich), 1% GlutaMAX (Gibco), and 1% Zellshield (Minerva Biolab, Skillman, NJ, USA).	/	Growth factor reduced matrigel	ICCA patient xenograft	Histological staining
Song et al. 2023	10.1007/s12072-023-10556-3.	10	ICC tissues-derived organoid medium composition: basal medium + human liver expansion medium (B27 supplement (without vitamin A) (50 × ; Life Technologies, cat. no. 12587-010), N2 supplement (100 × ; Life Technologies, cat. no. 17502-048), 1 mM N-acetylcysteine (Sigma-Aldrich, cat. no. A0737-5MG), 10% (vol/vol) Rspo1-conditioned medium, 10 mM nicotinamide (Sigma-Aldrich, cat. no. N0636), 10 nM recombinant human [Leu15]-gastrin I (Sigma-Aldrich, cat. no. G9145), 50 ng/ml recombinant human EGF (Peprotech, cat. no. AF-100-15), 100 ng/ml recombinant human FGF10 (Peprotech, cat. no. 100-26), 25 ng/ml recombinant human HGF (Peprotech, cat. no. 100-39), 10 μM Forskolin (Tocris Bioscience, cat. no. 2939) and 5 μM A83-01 (Tocris Bioscience, cat. no. 2939)). Liver tissues-derived organoid medium composition: 30% wnt3a-conditioned medium + basal medium + human liver expansion medium.	/	Matrigel	ICCA tissue	Histological staining, PCR
Li et al. 2024	10.1097/HC9. 0000000000000360	/	Complete medium including growth factors	/	Matrigel	ICCA tissue	Histological staining, mRNA and protein expression, growth curve, tumorigenicity through transplantation
Lieshout et al. 2023	10.1002/ijc.34350.	4	Organoid expansion medium supplemented with 2, 10, 50 or 200 ng/ml of cytokines IL-1β (Bio-Techne), IL-6 (Life Technologies Europe BV), IL-17A (Merck Life Science NV), IFNγ (Life Technologies Europe BV) and TNFα (Peprotech). A vehicle control (IL-1β, IL-6, IFNγ: PBS with 0.1% BSA; IL-17A: distilled water; TNFα: DMSO; diluted 500 times in culture medium) was included in every experiment.	/	Basement membrane extract	CCA tissue	Targeted NGS, tumorigenicity through transplantation
Boden et al. 2025	10.3390 /biomedicines13051083.	10 PSC organoids + 8 CCA organoids	DMEM/F12 Medium (500 ml), (2x) 1% Penicillin/Streptomycin, (2x) 1% GlutaMAX, (2x) 10 mM HEPES, (2x) 1:50 B27 supplement (without Vitamin A), (2x) 1:100 N2 supplement, (2x) 10 nM recombinant human [Leu15]-Gastrin I, (2x) 1.25 mM n-Acetyl-L-cysteine, (2x) 10 mM nicotinamide, R-Spondin1 conditionend media (25 ml), 50 ng/ml recombinant human EGF, 100 ng/ml recombinant human FGF10, 25 ng/ml recombinant human HGF, 10 μM Forskolin, 5 μM A8301, 100 μg/mL Normocin.	Maximum growth for PSC organoid was 13 passages. 8/14 CCA organoids were considered long-term (≥10 passages)	Geltrex matrix	Human CCA tissue and tissue from PSC patients	Histological staining
Frank et al. 2024	10.1007/s10620-024-08570-y	10 PSC organoids + 3 control organoids	Sampaziotis et al. 2017	/	Matrigel	Cholangiocytes from PSC and control patients	Histological staining, qPCR, single cell RNA sequencing
Kang et al. 2024	10.1002/advs. 202306174	/	ICC organoid complete medium (BioGenous, Hangzhou, China)	/	Matrigel	ICCA hepatolithiasis tissue	/
Ye et al. 2023	10.1371/journal.pone. 0283737	28 choledochal cyst organoids	Advanced DMEM/F12 (Invitrogen) supplemented with Penicillin/Streptomycin (Invitrogen), GlutaMax (Invitrogen), 25 mM HEPES (Invitrogen), 1% N2 (GIBCO), 1% B27 (GIBCO), 1.25 mM Acetylcysteine (Sigma), 10 nM gastrin (G9145; Sigma), 50 ng/ml EGF (PMG8043; PeproTech), 100 ng/ml FGF10 (100-26-25UG; PeproTech), 25 ng/ml HGF (100-39-10UG; PeproTech), 10 mM Nicotinamide (Sigma), 5 μM A83.01 (Tocris), 10 μM Forskolin (Tocris), 500 ng/ml R-Spondin 1 (7150-RS-025; R&D), 100 ng/ml Noggin (250-38-20UG; Peprotech), 100 ng/ml Wnt3a (1324-WN-010; R&D) and 250 ng/ml Amphotericin B (#15290018; GIBCO). For the first six days of culture, 10 μM ROCK inhibitor Y-27632 (Tocris).	/	Matrigel	Liver and bile duct biopsies of choledochal cysts	RNA sequencing
Nakagawa et al. 2017	10.1073/pnas. 1619416114	24	Ad-DMEM/F12 (Invitrogen) supplemented with B27 and N2, 1 mM N-acetylcysteine, 10 mM nicotinamide (Wako), 50 ng/mL EGF, 1 μg/mL Rspo1, 100 ng/mL Noggin, 100 ng/mL FGF10 (Peprotech), and 10% Wnt3a-conditioned medium (ATCC).	/	Matrigel	Murine extrahepatic bile duct tissue	Histological staining, western blot, PCR, Tumorigeneity through xenografting, cDNA microarray analysis

Razumilava et al. 2019	10.1002/hep4.1295	/	50% L-WRN (CRL-3276; ATCC, Manassas, VA) conditioned media,30 1X penicillin–streptomycin, 1X GlutaMAX, 10 mM 4-(2-hydroxyethyl)-1-piperazine ethanesulfonic acid, 1X Fungizone, 1X gentamicin, 1X B27, and 1X N2 (Thermo Fisher Scientific) in advanced Dulbecco's modified Eagle's medium/F12 (Invitrogen, Carlsbad, CA). Fibroblast growth factor 10 (100 ng/mL; PeproTech, Rocky Hill, NJ) and epithelial growth factor (50 ng/mL; Invitrogen) were added to the culture media for the first 3 days.	/	Matrigel	Murine extrahepatic bile duct tissue	mRNA expression
Roos et al. 2022	10.1016/j.stem. 2022.04.011.	20 intrahepatic cholangyocyte organoids (14 could be induced into branching organoids	Branching organoids were created by switching culture conditions to non-canonical WNT stimulating conditions, consisting of William's E medium (WE, ThermoFisher Scientific) supplemented with 10 mM nicotinamide (Sigma), 17 mM sodium bicarbonate (Sigma), 0.2 mM 2-phospho-L-ascorbic acid trisodium salt (Sigma), 6.3 mM sodium pyruvate (Gibco), 14 mM glucose (Sigma), 20 mM HEPES (Fisher Scientific), ITS + premix (Life Technologies), 0.1 µM dexamethasone (Sigma), 2 mM Ultraglutamine (Fisher Scientific), 100 µg/mL penicillin/streptomycin (Fisher Scientific), 20 ng/mL EGF (Peprotech), 10% RSPO1 conditioned media (homemade) and 100 ng/mL DKK-1 (Abcam Ltd), after three passages.	>20 passages	Matrigel	Adult and fetal liver (intrahepatic bile ducts) and adult extrahepatic bile ducts	Growth monitoring through microscopy, transmission electron microscopy, histological staining, qRT-PCR, single cell RNA sequencing, live imaging, western blot
Mi et al. 2025	10.1002/ijc.35483	3 undifferentiated CCA organoids + 3 matured branching phenotype CCA organoids	Expansion medium or branching medium (Roos et al. 2022)	/	Basement membrane extract	CCA tissue	Histological staining, proliferation rate, mRNA sequencing
van Tienderen et al. 2023	10.1016/j.bioadv. 2023.213289	3	Adv+ medium, N2, B27, N-Acetylcystein, gastrin, EGF, FGF10, HGF, nicotinamide, A83. 01, Forskolin, R-Spondin conditioned medium.	/	Hydrogel derived from decellularized tumor ECM or BME	CCA tissue	Broutier et al. 2017
Asim et al. 2024	10.1016/j.ijbiomac. 2024.130657.	1	/	7 days	Hydrogel with 1 % w/v gelatin, 5%GelMa and Matrigel (control)	HuCCT-1 cells	Growth monitoring, histological staining
Lidsky et al 2022	10.1038/s41698-022-00320-5	1	Advanced DMEM/F12, 1× GlutaMax, 10 mM HEPES, 1× Antibiotic–Antimycotic, 1× N2 supplement, 1× B27 supplement, 100 ng/mL human EGF, 1.25 mM N-acetylcysteine, 10 nM gastrin, 10 mM nicotinamide, 5 µM A83-01, 10 µM forskolin, 10 µM Y-27632, 10% Rspo-1 condition media, 100 ng/mL human FGF-10, 25 ng/mL human HGF.	/	Matrigel	PDX tissue	Histological staining, western blot
van Tienderen et al. 2022	10.3390/cells11223657	3 CCA organoids + 5 intrahepatic cholangiocyte organoids	Adv+ medium, N2, B27, N-Acetylcystein, gastrin, EGF, FGF10, HGF, nicotinamide, A83. 01, Forskolin 10, R-Spondin 10% Conditioned medium.	/	Microcapsules utilizing enzymatic outside-in crosslinking of cell-laden hydrogel precursor droplets or standard BME culture (control)	CCA tissue + donor liver biopsies	Growth monitoring though microscope, histological staining, real-time PCR
Wang et al. 2022	10.1038/s41420-022-01014-4	1 CCA cell line organoids + 4 CCA PDOs	Advanced DMEM/F12 (Gibco, CS, USA) supplemented with 1x penicillin/streptomycin (ThermoFisher, MA, USA), 1× Glutamax (ThermoFisher, MA, USA), 10 mM HEPES (ThermoFisher, MA, USA), 1× B27 supplement (Gibco, CS, USA), 1× N2 supplement (Gibco, CS, USA), 10 nM gastrin (Sigma, MO, USA), 5 µM A83-01 (Tocris, Bristol, UK), 10 µM Y-27632 (Tocris, Bristol, UK), 50 ng/mL recombinant human epidermal growth factor (EGF, PeproTech, NJ, USA), 100 ng/mL recombinant human fibroblast growth factor 10 (FGF10, PeproTech, NJ, USA), 500 ng/mL recombinant human R-Spondin1 (R-Spo1, PeproTech, NJ, USA) and 10% v/v Afamin/Wnt3a CM (MBL Life Science, Japan). 25 ng/mL recombinant human Noggin (PeproTech, NJ, USA) was added for the CCA organoids.	/	Growth factor reduced matrigel	Cell lines RBE and CCA tissue	Growth monitoring through microscope, western blot, histological staining, whole exome sequencing
Roalsø et al. 2025	10.1016/j.pan. 2024.12.018	1 dCCA organoid	Complete organoid growth media consisted of equal parts conditioned media and basal media, supplemented further with B27 1X (Thermo Fisher Scientific), nicotinamide 10 mM (Sigma-Aldrich), N-acetylcystein 1.25 mM (Sigma-Aldrich), EGF 50 ng/mL (PeproTech), FGF-10 100 ng/mL (PeproTech), gastrin 10 nM (Tocris Bioscience), A83-01 500 nM (Tocris Bioscience), and if recently passaged Y-27632 10.5 µM. Conditioned media was derived from LWRN cell (ATCC CRL-3276) producing Wnt3a, R-spondin 3 and noggin. Briefly, CRL-3276 cells were expanded in selection media made of DMEM (Thermo Fischer Scientific) and FBS 10 % (Biowest), further containing G418 0.5 mg/mL (Gibco) and Hygromycin-B 0.5 mg/mL (Gibco) and subsequently cultured in ADMEM/F12 supplemented with FBS 10 % and Penicillin-Streptomycin 1 %. The conditioned media was harvested, centrifuged, and sterile filtered. Basal media comprised Advanced DMEM/F12 medium (Thermo Fisher Scientific), supplemented with HEPES 10 mM (Thermo Fisher Scientific), GlutaMAX 1X (Thermo Fisher Scientific), Penicillin-Streptomycin 1 % (Biowest).	/	Cultrex Reduced Growth Factor Basement Membrane Extract, Type 2	Tissue from pancreatomies	Growth monitoring through microscope, histological staining, mutational profile analysis, STR analysis, biobanking efficacy

	Kinoshita et al. 2023	10.1016/j.labinv.2023.100105.	62 bile-derived organoids: 60 biliary cancer + 2 non-cancer organoids	Advanced Dulbecco modified Eagle medium/F12 (Thermo Fisher Scientific) supplemented with 1% penicillin/streptomycin, 1% glutamax, 10 mM 4-(2-hydroxyethyl)-1-piperazineethanesulfonic acid, 1% N2 supplement, 2% B27 supplement (all from Thermo Fisher Scientific), 1.25 mM N-acetyl cysteine, 10 nM gastrin, 10 mM nicotinamide (all from Sigma-Aldrich), 0.2% primocin (InvivoGen), 50 ng/mL EGF (PeproTech), 10% Rspo-1–conditioned medium (Trevigen), 5 μM A83-01 (Tocris Bioscience), 10 μM forskolin (Tocris Bioscience), and 10 μM Y-27632 (Sigma-Aldrich).	/	Matrigel	Tissue from endoscopic retrograde cholangiopancreatography	Morphology examination, sequencing, tumorigenicity through xenografting
	Kasuga et al. 2021	10.1111/cas.14703.	6	DMEM‐F12 supplemented with recombinant human hepatocyte growth factor (50 ng/mL, Biolegend), recombinant human epidermal growth factor (50 ng/mL; Pepro Tech), recombinant human fibroblast growth factor 10 (100 ng/mL, Biolegend), nicotinamide (10 mmol/L, Sigma‐Aldrich), recombinant human R‐spondin–1 (0.5 μg/mL, Miltenyi Biotec), B27 supplement without vitamin A (Life Technologies), and Y‐27632 (10 μmol/L).	/	Growth factor reduced matrigel	Growth factor reduced matrigel	Flow cytometric analysis, histological staining, tumorigeneisis through transplatation, secondary organoid formation, microarray
	Inoue et al. 2022	10.3390 /biomedicines10123133	/	DMEM‐F12 supplemented with recombinant human hepatocyte growth factor (50 ng/mL, Biolegend), recombinant human epidermal growth factor (50 ng/mL; Pepro Tech), recombinant human fibroblast growth factor 10 (100 ng/mL, Biolegend), nicotinamide (10 mmol/L, Sigma‐Aldrich), recombinant human R‐spondin–1 (0.5 μg/mL, Miltenyi Biotec), B27 supplement without vitamin A (Life Technologies), and Y‐27632 (10 μmol/L).	/	Growth factor reduced matrigel	Growth factor reduced matrigel	Flow cytometric analysis, histological staining, tumorigeneisis through transplatation, secondary organoid formation, microarray (organoids previously established and described in Inoue et al. 2022)
	Chen et al. 2024	10.1016/j.canlet.2023.216586	1 CCA	Advanced DMEM/F12, 1x GlutaMax, 1x B27, 10 mM Nicotinaminde, 1.25 mM N-Acetyl Cysteine, 20 mM HEPES, 10 nM Gastrin, 5 ng/mL FGF-basic, 15 ng/mL FGF-10, 50 ng/mL EGF, 100 ng/mL Wnt3a, 200 ng/mL Noggin, 250 ng/mL R-spondin-1, 500 nM A83-01, 10 μM SB202190, and 10 μM Y-27632.	/	Growth factor reduced Matrigel	CCA tissue	Growth monitoring through microscope, histological staining, single cell RNA sequencing
	Piansaddhayanon et al. 2023	10.1038/s41597-023-02482-8	/	Advanced DMEM/F12 containing 10% R-Spondin condition media, 10% Wnt3a condition media, 1 mM N-Acetylcysteine, 10 mM Nicotinamide, 1x B27 supplement, 1x N2 supplement, 100 ng/ml Noggin, 10 nM Gastrin-I, 50 ng/ml EGF, 5 uM A83-01, 100 ng/ml FGF10 (Peprotech), 25 ng/ml HGF (R&D Systems), and 10 μM FSK (Tocris).	/	Matrigel	Liver tissue from CCA patients	Fluorescent labelling of dissociated organoid cells
**Co-culture**								
	Okabe et al. 2011	10.1245/s10434-010-1391-7	2	RPMI or DMEM with 10% FBS and penicillin/streptomycin; HUVECs cultured with EGM-2 kit; CM prepared in serum-free DMEM.	12h/48h depending on the assay	Matrigel for tube formation assay	Cell lines HuCCT-1, RBE (cholangiocarcinoma), LI90, LX-2 (hepatic stellate cells), HUVECs	Immunohistochemistry, Western blot, TUNEL apoptosis assay, Matrigel invasion assay, tube formation
	Raggi et al. 2017	10.1016/j.jhep.2016.08.012	2 conditioned media	Selective serum-free Dulbecco's modified Eagle's medium (DMEM)/F12 medium supplemented with 1X B27 supplement minus vitamin A (Life Technologies), human recombinant epidermal growth factor (hrEGF) (R&D System) (20 ng/ml), and bFGF (R&D System) (20 ng/ml).	15 days	Anchoring-independent conditions	Established (SG231, HUCCT1, CCLP1) and primary human intrahepatic CCAderived (CCA4) cell lines; freshly isolated healthy donor circulating monocytes (CD14+)	Chemotaxis chamber assay, FACS, cell proliferation, tube formation
	Sueca-Comes et al. 2024	10.1242/dmm.050716	3 spheroids + 7 PDX spheroids	High-glucose Dulbecco's modified essential medium (DMEM) supplemented with 1 mM L-glutamine and 10% heat-inactivated foetal bovine serum (10% DMEM).	/	Basement membrane extract/Matrigel	KKU-M055, KKU-M213 and RBE cell lines, PDX tumors and immortalized mesenchymal stem cells	Growth monitoring through microscopy, live/dead staining, histological staining, differential expression analysis
	Gondaliya et al. 2024	10.1016/j.ymthe.2024.06.006	2	DMEM-HG with 10% FBS & 1% Pen-Strep was used for all tumor, LX2, SB cells. For DF & HUVEC, supplier-recommended media was utilized.	3 days	Methylcellulose	Human cell lines: HuCCT1, SNU1079, RBE, CCLP (cholangiocarcinoma); LX2 (hepatic stellate); SB (cholangiocyte); DF (dermal fibroblasts); HUVEC	Growth observation through microscopy, live/dead cell imaging, PCR
	Tian et al. 2023	10.1038/s41388-023-02639-0.	3	/	/	/	Tumors collected from a metastatic iCCA orthotopic allograft model, intratumoral myofibroblasts, peritumoral myofibroblasts	Growth monitoring through microscopy, quantification of spheroid diameter
	Manzanares et al. 2017	10.1016/j.ajpath.2017.01.013	1	DMEM + 100 U/mL Penicillin, 100 μg/mL Streptomycin, 0.1 μmol/L Insulin, 5 μg/mL Transferrin, 10% FBS (erste 4-24h), later 1% FBS	/	Rat tail type I collagen	Rat cancer-associated myofibroblasts and a pure rat cholangiocarcinoma cell strain	Picrosirius red staining, histological staining, western blot
	Campbell et al. 2012	10.1111/j.1872-034X.2012.01026.x.	1	Standard medium with TGF-β1 (5 ng/ml) to facilitate CAF expansion	10 days	Rat tail type I collagen gel	Cholangiocarcinoma cell line (BDEsp-TDE) and the corresponding tumor-derived α-SMA positive CAF cell line (BDEsp-TDF).	Histological staining, picrosirius red staining, invasion assay

	Liu et al. 2014	10.1002/hep.27085	1	Standard medium with 1% FBS	8 days	Rat tail Type I collagen gel for organotypic co-culture model; Matrigel-coated chambers for invasion assay	Tumor-derived BDEsp-TDEH10 CCA cell strain, and tumor-derived BDEsp-TDFE4 cancer-associated myofibroblastic cell strain	Campbell et al. 2012
	Boonsri et al. 2021	10.4143/crt.2020.585	/	Modified RPMI-1640 (phenol red-free, pH 6.8) + 6 mM D-glucose	/	Cultrex basement membrane extract	CCA cells cultivated with CAFs from human tissue or human bone-marrow-derived mesenchymal stem cells	Growth and cell survival measurements
	Aoki et al. 2022	10.1136/gutjnl-2020-322493.	1	Standard medium with 4% FBS	7 days (spheroid formation)	Agarose-coated wells, then transferred onto Matrigel	Mouse ICC cell lines + primary quiescent murine CAFs (from murine ICC tumors)	/
	Schrom et al. 2023	10.3390 /cancers15061757.	1	DMEM/F12 + 10% FBS + 2 mM L-Glutamine + 1× Pen/Strep	6 days	Ultra-low attachment plates	Primary human tumor cells (MUG CCArly) from patient tumor tissue + immortalized CAF (CCArly CAF) from same patient using hTERT	Growth monitoring through microscopy, live/dead staining, mass spectrometric analysis
	Mancarella et al. 2024	10.1186/s13046-024-03210-9.	6	For co-culture, it was used Complete Dulbecco's modified Eagle medium (DMEM), (Gibco, Grand Island, NY, USA) supplemented with 10% Fetal Bovine Serum (FBS), Antibiotic-Antimycotic, Sodium Pyruvate and Hepes, (Gibco, Grand Island, NY, USA). Complete IMDM (Iscove's Modified Dulbecco's Medium) with 20% FBS and Antibiotic–Antimycotic for CAF culture.	3 days (formation) + up to 5 days (treatment)	Engineered hydrogel (bovine skin collagen I + Matrigel, pH neutralized with 0.5 M acetic acid)	iCCA cell lines: KKU-M213 and KKU-M156 (human, from JCRB/ATCC); hCAFs: isolated from 3 fresh iCCA human patient tissues (primary)	Histological staining
	Li et al. 2023	10.1016/j.isci. 2023.106095	2	Li et al. 2019; Massani et al. 2013	14 days	Matrigel	Fibroblasts and epithelial cancer cells from CCA tissue	Histological staining, live imaging, qRT-PCR
	Guo et al. 2024	10.1016/j.heliyon.2024. e36377	6	Advanced DMEM/F12 supplemented with 1 % penicillin/streptomycin (1 × , ThermoFisher, MA, USA), Glutamax (1 × ; ThermoFisher), B27 supplement (1 × ; Gibco, CS, USA), N2 supplement (1 × ; Gibco, CS, USA), HEPES (10 mM, ThermoFisher, MA, USA), gastrin (10 nM; Sigma Aldrich, MO, USA), A83-01 (5 μM; Tocris, Bristol, UK), Y-27632 (10 μM; Tocris, Bristol, UK), recombinant human epidermal growth factor (50 ng/mL; PeproTech, NJ, USA), recombinant human fibroblast growth factor 10 (100 ng/mL; PeproTech), recombinant human R-Spondin1 (500 ng/mL; PeproTech), recombinant human Noggin (100 ng/mL; PeproTech), and Afamin/Wnt3a CM (10 % v/v; MBL Life Science, TKY, Japan).	/	Growth factor reduced Matrigel	Cancer cells from CCA tissue and Peripheral blood mononuclear cells isolated from peripheral blood samples of patients with CCA	Growth observation through microscopy, histological staining, whole exosome sequencing
	Zhou et al. 2022	10.1038/s41416-022-01839-x	/	Organoid expansion medium, supplemented with 0, 2.5, 5, 7.5, or 10% human serum, supplemented with 0, 20 IU/ml (4 ng/ml) or 100 IU/ml (20 ng/ml) IL-2 or deprived of nicotinamide and/or forskolin for 6–7 days.	/	Basement membrane extract	CCA tissue, peripheral blood monocytes, T cellls	Growth observation through microscopy, flow cytometry
	van Tienderen et al. 2023	10.1016/j.actbio. 2022.11.038.	/	Adv+ medium, N2, B27, N-Acetylcystein, gastrin, EGF, FGF10, HGF, nicotinamide, A83. 01, Forskolin, R-Spondin conditioned medium.	7 days	Decellularized CCA tissue, decellularized tumor-free liver tissue or basement membrane extract (control)	CCA tissue	Growth observation through microscopy, quantification of the DNA content, bulk RNA sequencing
**Spheroids**								
	Cardinale et al. 2015	10.1016/j.ajpath. 2015.02.010	2	serum-free medium of DMEM with high glucose/DMEM:F12 mixture (1:1) (Gibco/BRL; Life Technologies) supplemented with 20 ng/mL EGF, 10 ng/mL FGF-2, and 1× B27 (Gibco/BRL; Life Technologies)	7 days	Ultra Low Attachement	human cell lines of IH- and pCCA, CCA and peritumoral noncancerous liver tissue	Light microscopy, Immunofluorescence
	Arnoletti et al. 2018	10.1080/15384047. 2018.1480292	3	rich medium with or without the presence of supplements reported to support cluster formation and fibroblast differentiation from myeloid cells: 50% Matrigel, 10 μg/ml human fibronectin, 10 μg/ml human adiponectin, or 0.5 μg/ml macrophage colony stimulating factor (MCSF)	7 or more days	Matrigel	CTC from PDAC, CC, and AA patients	photo-microscopy, DNA and mRNA analysis
	Panawan et al. 2023	10.1111/cas.15812	2	/	more than 3 months	Ultra Low Attachement	CCA cell line	Histological staining, stem cell marker expression
	Wang et al. 2023	10.1007/s12015-023-10557-7	2	/	7 days	Ultra Low Attachement	CCA cell line	Flow Cytometry
	Yogo et al. 2023	10.1111/cas.15676	2	serum-free DMEM/Ham's F-12 (Nacalai Tesque). DMEM was supplemented with 1× B27 Supplement (Thermo Fisher Scientific), 10 ng/mL recombinant human fibroblast growth factor-basic (Thermo Fisher Scientific), 20 ng/mL recombinant human epidermal growth factor (EGF; Thermo Fisher Scientific), and 1% penicillin/streptomycin mixed solution (P/S; Nacalai Tesque)	5-7 days	ultra Low Attachement	BDC cell lines	qRT-PCR
	Huang et al. 2022	10.1186/s13287-022-02988-9	4	DMEM/F12 was supplemented with 20 ng/ml epidermal growth factor (EGF, Invitrogen), 10 ng/ml basic fibroblast growth factor (bFGF, Invitrogen), B27 (1:50; Invitrogen), N2 (1:100; Invitrogen), 1% sodium pyruvate, 100 μg/ml penicillin G and 100 U/ml streptomycin	7 days	ultra Low Attachement	CCA cell lines	mRNAsi expression, Tissue microarray (TMA) and immunohistochemistry (IHC)
	Yang et al. 2023	10.1002/mc.23589	2	serum-free DMEM-F12 (Gibco), EGF (20 ng/mL; PeproTech), bFGF (20 ng/mL; PeproTech), and B-27 Serum-Free Supplement (Gibco)	14 days	ultra Low Attachement	CCA tissue	qRT-PCR, Immunohistochemistry

Zhu et al. 2023	10.1007/s10735-023-10150-9	1	DMEM/F12 medium supplemented with B27 (Gibco), 20 ng/mL epidermal growth factor (Gibco), 20 ng/mL basic fibroblast growth factor (Nowoprotein, China) and 4 g/mL insulin (Gibco)	12–18 days	/	CCA cell lines	mRNA expression
Mannini et al. 2025	10.1111/liv.16208	2	serum-free DMEM/F12 medium supplemented with 1X B27 supplement without vitamin A (Life Technologies), 20 ng/mL EGF, and 20 ng/mL bFGF (R&D Systems)	7 days	anchoring-independent conditions	BDC tissue	chromatography, immunofluorescence
Peng et al. 2013	10.1038/bjc.2013.655	1	serum-free DMEM-F12 (Hyclone, Logan, UT, USA), supplemented with B27 (1 : 50; Invitrogen, Carlsbad, CA, USA), 20 ng ml−1 epidermal growth factor (PeproTech EC, London, UK), 100 ng ml−1 basic fibroblast growth factor (PeproTech EC), and 100 ng ml−1 leukaemia inhibitory factor (Chemicon, Billerica, MA, USA)	20 days	/	CCA tissue	marker expression
Qiu et al. 2019	10.3892/ijo.2019.4798	2	Dulbecco's modified Eagle's/F12 medium supplemented with 2% B27 (both from Gibco; Thermo Fisher Scientific, Inc.), 20 ng/ml epidermal growth factor (Sigma-Aldrich; Merck KGaA), 20 ng/ml basic fibroblast growth factor (Gibco; Thermo Fisher Scientific, Inc.) and 4 µg/ml heparin (Sigma-Aldrich; Merck KGaA)	10 days	/	CCA cell lines	long non-coding RNA expression profiling, fluorescence
Shi et al. 2024	10.1002/jgm.3689	4	/	10-14 days	ultra Low Attachement	ICC tissue	Three bulk and single-cell RNA-seq
Sugiura et al. 2019	10.1016/j.ajpath.2019.05.014	2	/	7 days	ultra Low Attachement	ICC cell lines	anoikis assay
Roncoroni et al. 2018	10.1080/01635581.2018.1470648.	2	Iscove Modified Dulbecco’s Medium (IMDM–GIBCO, Italy), added with 1% penicillin 100 U/mL streptomycin 100 mg/mL (GIBCO, Italy), 2% L-glutamine 200 mM (GIBCO, Italy)	7 days		CCA cell lines	light microscopy, transmission electron microscopy
Pant et al. 2022	10.3389/fcell.2021.809382	1	/	/	/	CCA cell lines	IncuCyte
Pang et al. 2022	10.1016/j.bioorg.2022.105679	2	DMEM/F-12 supplemented with B27 (Life Technologies, USA, 1:50), N2 (Life Technologies, 1:100), 20 ng/ml epidermal growth factor (EGF), 10 ng/ml bFGF, 100 units/ml penicillin, and 100 ng/mL streptomycin. And DMSO or 30 µM different glycoside analogues were added in the tumor sphere medium.	7-9 days	ultra Low attachement	CCA cell lines	Drug screening
Myint et al. 2024	10.3390/ph17020197	1	10% FBS supplemented DMEM-high glucose medium	10 days	Matrigel	CCA cell lines	qRT-PCR, SDS-PAGE and Immunoblotting
Sittithumcharee et al. 2019	10.1002/hep.30704	2	culture media containing 2.5% Matrigel	3 days	Matrigel	CCA cell lines	protein and gene expression profiles
Sungwan et al. 2021	10.7717/peerj.11067	1	Dulbecco’s Modified Eagle’s Medium (DMEM) (Gibco life technologies Corporation, Grand Island, NY) with 25 mM glucose supplemented with 1% antimycotic-antibiotic and 10% heat-inactivated fetal bovine serum	24-72h	ultra Low attachement	Human CCA cell lines derived from CCA patient tissues	Differentially expressed gene (DEGs) analysis
Lu et al. 2021	10.1038/s41419-020-03346-4	2	DMEM/F12 (Gibco, USA) supplemented with 2% B27, 10 ng/ml EGF and 10 ng/ml FGF	/	ultra Low attachement	frozen cholangiocarcinoma and paired adjacent normal bile duct tissue + CCA cell lines	in situ hybridization analysis
Namikawa et al. 2023	10.1002/path.6139.	2	DMEM (FUJIFILM Wako Pure Chemical Corp., Osaka, Japan), 10% FBS, 1 mg/ml collagenase D (Roche Diagnostics Deutschland GmbH, Mannheim, Germany), 0.5 mg/ml dispase (Invitrogen), and 40 µg/ml DNase (Roche Diagnostics Deutschland GmbH)	/	Matrigel	CCA cell lines	/
Yang et al. 2021	10.1016/j.aohep.2020.09.009	3	Dulbecco's Modified Eagle Medium/Nutrient Mixture F-12 (DMEM/F12) medium (Invitrogen, USA), supplemented with 1 × B-27 Serum-Free Supplement (Invitrogen, USA), 4 µg/mL heparin (Stem cell Technologies, Canada), 100 U/mL penicillin and 100 µg/mL streptomycin, 10 ng/mL epidermal growth factor (EGF) (Invitrogen, USA), and 10 ng/mL basic fibroblast growth factor (bFGF) (Invitrogen, USA)	6 days	ultra Low attachement	CCA cell lines	real-time RT-PCR
Olaizola et al. 2025	10.1097/HEP.0000000000001259	3	/	3 days	/	CCA cell lines	/
Gentilini et al. 2019	10.1016/j.bbadis.2019.04.020	2	selective serum-free DMEM/F12 medium supplemented with 1× B27 supplement minus vitamin A (Life Technologies), 20 ng/ml human recombinant epidermal growth factor, and 20 ng/ml bFGF (R&D System)	15 days	ultra Low attachement	CCA cell lines	RTQ-PCR, Western blot analysis
Zhu et al. 2022	10.1111/liv.15462	2	serum-free DMEM-F12 (Gibco, USA), EGF (20 ng/mL, PeproTech, USA), bFGF (20 ng/mL, PeproTech) and B-27 Serum-Free Supplement (Gibco)	14 days	Ultra-Low Attachment	CCA tissue	flow cytometry
Vaquero et al. 2018	10.1158/1078-0432.CCR-17-3725	4	serum-free DMEM/F12 medium, supplemented with 100 mg/mL gentamycin (Sigma-Aldrich), B27 (Life Technologies), 20 ng/mL human epidermal growth factor (EGF, Life Technologies), 20 ng/mL human basic fibroblast growth factor (bFGF, Life Technologies), and 1% antibiotic–antimycotic solution (Life Technologies)	7 days	ultra Low attachement	CCA tissue	Microarray hybridization, RT-qPCR, Western Blot analysis
Mayr et al. 2023	10.1371/journal.pone.0287769	3	high-glucose Dulbecco’s modified Eagle’s medium (DMEM, Gibco, ThermoFisher Scientific, Waltham, MA, USA) supplemented with 10% (v/v) fetal bovine serum (FBS, Biochrom, Berlin, Germany), 1% antibiotic–antimycotic (ABAM, Merck, Darmstadt, Germany), 1 mM sodium pyruvate (Pan Biotech, Aidenbach, Germany), and 10 mM HEPES (Pan Biotech)	8 days	ultra Low attachement	BTC cell lines	mRNA expression
Song et al. 2024	10.1016/j.phymed.2024.155944	4	DMEM/F-12 medium, 0.1% collagenase type IV, 0.05% hyaluronidase, and 0.01% deoxyribonuclease	5–7 days	microfluidic chip	CCA tissue	Immunohistochemistry, AO/PI staining
Kawamoto et al. 2018	10.21873/anticanres.12516	2	serum-free DMEM/F12 containing 20 ng/ml epidermal growth factor (Invitrogen, Grand Island, NY, USA), and 20 ng/ml basic fibroblast growth factor (Invitrogen, Seoul, Korea) [DMEG(+)GF], high glucose DMEM with FBS [DMEM(+)FBS], or high glucose DMEM without FBS [DMEM(−)FBS]	7 days	ultra Low attachement	CCA cell lines	Western Blot analysis

Miao et al. 2022	10.1186/s12935-022-02840-3	1	serum-free conditioned stem cell medium (RPMI-1640 medium supplemented with 1 × B27, 20 ng/mL human EGF, 10 ng/mL human FGF, 0.4% bovine serum albumin, and 4 μg/mL insulin)	14 days	ultra Low attachement	ICC tissue	Immunohistochemistry, RNA sequencing
Cavalloni et al. 2016	10.1007/s13277-015-4215-3.	1	stem cell medium serum free (SC medium: DMEM-F12 medium, 1× B27, 200 ng/mL human EGF, 10 ng/mL human FGF, 0.4 % BSA, 4 μg/mL insulin and P/S)	14 days	ultra Low attachement	ICC tissue	Immunohistochemistry
Fu et al. 2022	10.1016/j.hbpd. 2022.03.006	2	DMEM/F12 medium supplemented in addition with 1X B27 supplement (Gibco) and human recombinant epidermal growth factor (hrEGF) (Gibco) (20 ng/mL), and bFGF (Gibco) (10 ng/mL)	7 days	ultra Low attachement	ICC cell lines	Western Blot analysis, Flow cytometry
Bai et al. 2022	10.31083/j.fbl2701018	4	DMEM/F-12 supplemented with B27 (Life Technologies, USA, 1:50), N2 (Life Technologies, 1:100), 20 ng/mL epidermal growth factor (EGF), 10 ng/mL bFGF, 100 units/mL penicillin, and 100 ng/mL streptomycin and seven preliminary screening small molecules with a concentration of 10 μM	5 days	ultra Low attachement	ICC cell line	Immunohistochemistry, Western Blot analysis, qRT-PCR
Menapree et al. 2025	10.1038/s41598-025-90997-y	7	HAM's F-12 medium (HyClone Laboratories, Logan, Utah, USA)	18 days	ultra Low attachement	CCA cell line	calcein AM and propidium iodide (PI) fluorescence
Phanthaphol et al. 2021	10.3389/fonc. 2021.657868	3	Dulbecco's Modified Eagle's Medium (DMEM) or in DMEM/F12 (Gibco; Invitrogen Corporation, Carlsbad, CA, USA) supplemented with 10% fetal bovine serum (FBS), penicillin (100 U/ml), and streptomycin (0.1 mg/ml)	5 days	ultra Low attachment with 2.5% Corning matrigel matrix	CCA cell lines	propidium iodide (PI) fluorescence
Phanthaphol et al. 2025	10.1186/s12967-025-06453-y	3	Dulbecco's Modified Eagle's Medium (DMEM) /F12 (Gibco; Thermo Fisher Scientific, Waltham, MA, USA) with 10% heat-inactivated fetal bovine serum (FBS, Gibco; Invitrogen) and 100 μg/ml penicillin/streptomycin (Sigma-Aldrich Corporation, St. Louis, MO, USA)	5 days	ultra Low attachment with 3% Corning Matrigel matrix	CCA cell lines	propidium iodide (PI) fluorescence
Sangsuwannukul et al. 2020	10.1016/j.intimp. 2020.107069	3	/	5 days	Matrigel, ultra Low attachment	CCA cell lines	Brightfield and Wasabi green fluorescent images
Supimon et al. 2023	10.1016/j.jcyt. 2022.10.006	4	/	48h + 5 days co-culture	Matrigel, ultra Low attachement	CCA cell lines	fluorescence
Suwanchiwasiri et al. 2024	10.1016/j.biopha. 2024.116718	2	Dulbecco's Modified Eagle's Medium (DMEM) F12 (Gibco; Thermo Fisher Scientific, Waltham, MA, USA) supplemented with 10% heat-inactivated fetal bovine serum (FBS), 100 U/ml of penicillin, and 0.1 mg/ml of streptomycin	2 days	ultra Low attachment with 3% Corning Matrigel matrix	CCA cell lines	propidium iodide (OI) fluorescence
Thongsin et al. 2024	10.1186/s13287-024-04029-z.	2	RPMI-1640 (Gibco™), 10% FBS, 2 mM GlutaMAX™, 1% non-essential amino acid (Gibco™), and 1% penicillin/streptomycin supplemented with 1 μg/ml propidium iodide (PI)	5 days	ultra Low attachement	CCA cell lines	propidium iodide (OI) fluorescence
Hasegawa et al. 2024	10.1111/cas.16306	2	DMEM/F12 (Fujifilm Wako Pure Chemical Corporation) supplemented with B27 (1:50; Thermo Fisher Scientific), epidermal growth factor (20 ng/mL; PeproTech), and fibroblast growth factor-2 (20 ng/mL; PeproTech)	7 days		CCA PDX cell line	scratch assay
Correnti et al. 2022	10.1111/liv.15049	4	selective serum-free Dulbecco's modified Eagle's medium (DMEM)/F12 medium supplemented with 1X B27 supplement minus vitamin A (Life Technologies), human recombinant epidermal growth factor (hrEGF) (R&D System) (20 ng/ml), and bFGF (R&D System) (20 ng/ml).	10 days		CCA cell lines	transfection of SB3 gene or addition of recombinant SB3 to cell medium, immunohistochemistry
Singsuksawat et al. 2018	10.1186/s12935-018-0525-z	2	Ham's F12 nutrient mixture (Invitrogen, Carlsbad, CA, USA) supplemented with 10% fetal bovine serum (FBS; Invitrogen).	15 days	ultra Low attachement	CCA cell lines	RT-PCR, immunohistochemistry
Carotenuto et al. 2017	10.1136/gutjnl-2016-312278	2	Dulbecco's Modified Eagle Media with 10% fetal bovine serum (FBS)	7 days	ultra Low attachement	CCA cell lines	microarray, real-time PCR and in situ hybridisation
Lobe et al. 2021	10.1002/hep.32069	2	/	/	/	/	/
Huang et al. 2025	10.1016/j.bbadis. 2025.167814	1	RPMI 1640 with 20 % FBS, 40 nM T3, and 20 μM gemcitabine	14 days	1.2 % melt agarose gel	CCA cell lines	stained with 0.005 % crystal violet solution
Yoshino et al. 2020	10.1093/carcin/bgz179	2	selective serum-free advanced Dulbecco's modified Eagle's medium (DMEM)/F12 medium (Thermo Fisher Scientific) supplemented with 1× B27 supplement minus vitamin A (Life Technologies), human recombinant epidermal growth factor (hrEGF) (R&D System) (20 ng/ml), and basic fibroblast growth factor (bFGF) (R&D System) (20 ng/ml)	15 days	ultra Low attachement	CCA cell lines	Knockdown and overexpression analysis
Shu et al. 2021	10.1038/s41419-021-04263-w.	2	selective serum-free advanced Dulbecco's modified Eagle's medium (DMEM) / F12 (Thermo Fisher Scientific, Waltham, MA, USA) supplemented with 2% B-27 supplement without vitamin A (Life Technologies, Carlsbad, CA, USA),	15 days	ultra Low attachement	human iCCA cell lines	Immunofluorescence staining, Western blotting
Ursu et al. 2019	10.1038/s41389-019-0153-z	1	DMEM-F12 media without fetal bovine serum, 1xB27 (Thermofisher Scientific), 20 ng/mL EGF, 20 ng/mL FGF (Peprotech, Rocky Hill, NJ), heparin, and 1× penicillin/streptomycin (Thermofisher Scientific)	/	/	CCA tissue	RT-PCR
Xie et al. 2018	10.1038/s41419-018-0286-6	4	serum-free DMED-12 (Hyclone, Logan, UT, USA), supplemented with B27 (1:50; Invitrogen, Carlsbad, CA, USA), 20 ng/ml epidermal growth factor (PeproTech EC, London, UK), 100 ng/ml basic fibroblast growth factor (PeproTech EC), and 100 ng/ml leukemia inhibitory factor (Chemicon, Billerica, MA, USA)	25 days	collagen IV	CCA cell lines	RT-PCR
Puthdee et al. 2022	10.1038/s41417-021-00387-5	3	DMEM/F12 supplemented with Gluta max, antibiotic-antimycotic B27, 20 ng/ml human recombinant epidermal growth factor (hEGF) and human basic fibroblast growth factor (hbFGF)	1 week	ultra Low attachement		RT-qPCR
Romanzi et al. 2023	10.3390 /biomedicines12010087	4	serum-free DMEM/F12 (Gibco, Thermo Fisher Scientific, Waltham, MA, USA) with 1x B27 (Gibco, Thermo Fisher Scientific, Waltham, MA, USA), 10 ng/mL EGF (Merck Life Science S.r.l., Milan, Italy), 10 ng/mL bFGF (Life Technologies, Waltham, MA, USA), and 1% Penicillin-Streptomycin	3 days	polyhydroxyethylmethacrylate (polyHEMA) 30 mg/mL (Merck Life Science S. r.l., Milan, Italy)	CCA cell lines	viability assay, Migration assay and Western blot, Immunofluorescence analysis

	Phukhum et al. 2023	10.1038/s41598-023-30204-y	3	Dulbecco's modified Eagle's medium (DMEM) supplemented with 10% FBS and 1% penicillin/streptomycin (Thermo Fischer Scientific, Massachusetts, USA)	48h	agarose-coating	CCA cell lines	Western blot analysis
	Mischiati et al. 2015	10.1371/journal.pone.0118906	2	/	7 days	/	CCA cell lines	mass spectrometry, Western blot analysis
	Raggi et al. 2017	10.1038/s41598-017-17804-1	3	selective serum-free DMEM/F12 medium supplemented with 1X B27 supplement minus vitamin A (Life Technologies, Monza, Italy), human recombinant EGF (R&D System, Milano, Italy) (20 ng/mL) and bFGF (R&D System) (20 ng/mL)	15 days	anchoring-independent conditions	CCA cell lines	Real-Time PCR
	Raggi et al. 2017	10.1016/j.jhep.2016.08.012	4	selective serum-free Dulbecco's modified Eagle's medium (DMEM)/F12 medium supplemented with 1X B27 supplement minus vitamin A (Life Technologies), human recombinant epidermal growth factor (hrEGF) (R&D System) (20 ng/ml), and bFGF (R&D System) (20 ng/ml)	15 days	anchoring-independent conditions	CCA tissue	In vivo experiments
	Raggi et al. 2021	10.1016/j.jhep.2020.12.031	2	selective serum-free DMEM/F12 medium supplemented with 1X B27 supplement without vitamin A (Life Technologies), 20 ng/mL EGF, and 20 ng/mL bFGF (R&D Systems)	7 days	anchoring-independent conditions	CCA tissue	Bioenergetic profiling, RT-PCR, Western Blot analysis
	Lori et al. 2024	10.1016/j.jhepr.2024.101182	2	selective serum-free DMEM/F12 medium supplemented with 1X B27 supplement without vitamin A (Life Technologies), 20 ng/mL EGF, and 20 ng/mL bFGF (R&D Systems) Technologies), 20 ng/mL EGF, and 20 ng/mL bFGF (R&D Systems)	7 days	anchoring-independent conditions	CCA cell lines	Liquid chromatography–mass spectrometry
	Di Matteo et al. 2019	10.1371/journal.pone.0210077	2	serum-free medium of DMEM with high glucose / DMEM:F12 mixture (1:1) (Gibco/BRL; Life Technologies) supplemented with 20 ng/mL EGF, 10 ng/mL FGF-2, and 1× B27 (Gibco/BRL; Life Technologies)	10 days	non-attached conditions	iCCA tissue	light microscopy
	Ciufolini et al. 2024	10.3390/cells13181536	2	DMEM High glucose (Gibco™-Thermo Fisher Scientific): Ham's F-12 Nutrient Mix ratio 1:1, B27, 2 mM glutamine, 20 ng/mL EGF, 20 ng/mL bFGF, Matrigel Corning 354234 2.5%	5 days	ultra Low attachement	CCA cell lines	brightfield fluorescence microscopy
	Yang et al. 2022	10.5114/ceh.2022.114192	4	DMEM/F12 medium, supplemented with 1 × B-27 serum-free supplement, 4 µg/ml heparin, 100 U/ml penicillin, 100 µg/ml streptomycin, 10 ng/ml EGF, and 10 ng/ml bFGF	6 days	ultra Low attachement	CCA cell lines	flow cytometry
	Xu et al. 2018	10.1016/j.prp.2018.09.005	2	serum-free DMEM/F12 medium (GIBCO, Grand Island, NY, USA) containing B27 supplement (GIBCO), 100 IU/ml penicillin, 100 µg/ml streptomycin, 20 ng/ml human recombinant epidermal growth factor (GIBCO), 10 ng/ml human recombinant basic fibroblast growth factor (GIBCO), 2% B27 supplement without vitamin A, 1% N-2 supplement (GIBCO), and 1% methyl cellulose (Sigma-Aldrich, St. Louis, MO, USA)	14 days	ultra Low attachement	CCA cell lines	Whole Human Genome Microarray Kit
	Kim et al. 2021	10.1186/s13071-021-04717-2	1	Dulbecco's Modified Eagle Medium (DMEM): DMEM/F12 (1:1) containing 10% FBS, an antibiotic mixture, 1.8 × 10−4 M of adenine, 5 µg/ml of insulin, 5.5 × 10−6 M of epinephrine, 2 × 10−9 M of triiodothyronine, 5 µg/mL of transferrin, 1.64 × 10−6 M of epidermal growth factor (EGF), and 1.1 × 10−6 M of hydrocortisone	5 days	SpheroFilm microwells	CCA cell lines	Microarray and RNA-Seq analysis
